# Metal A and Metal B Sites of Nuclear RNA Polymerases Pol IV and Pol V Are Required for siRNA-Dependent DNA Methylation and Gene Silencing

**DOI:** 10.1371/journal.pone.0004110

**Published:** 2009-01-01

**Authors:** Jeremy R. Haag, Olga Pontes, Craig S. Pikaard

**Affiliations:** Department of Biology, Washington University, St. Louis, Missouri, United States of America; Temasek Life Sciences Laboratory, Singapore

## Abstract

Plants are unique among eukaryotes in having five multi-subunit nuclear RNA polymerases: the ubiquitous RNA polymerases I, II and III plus two plant-specific activities, nuclear RNA polymerases IV and V (previously known as Polymerases IVa and IVb). Pol IV and Pol V are not required for viability but play non-redundant roles in small interfering RNA (siRNA)-mediated pathways, including a pathway that silences retrotransposons and endogenous repeats via siRNA-directed DNA methylation. RNA polymerase activity has not been demonstrated for Polymerases IV or V in vitro, making it unclear whether they are catalytically active enzymes. Their largest and second-largest subunit sequences have diverged considerably from Pol I, II and III in the vicinity of the catalytic center, yet retain the invariant Metal A and Metal B amino acid motifs that bind magnesium ions essential for RNA polymerization. By using site-directed mutagenesis in conjunction with *in vivo* functional assays, we show that the Metal A and Metal B motifs of Polymerases IV and V are essential for siRNA production, siRNA-directed DNA methylation, retrotransposon silencing, and the punctate nuclear localization patterns typical of both polymerases. Collectively, these data show that the minimal core sequences of polymerase active sites, the Metal A and B sites, are essential for Pol IV and Pol V biological functions, implying that both are catalytically active.

## Introduction

The largest and second-largest subunits of eukaryotic multi-subunit nuclear RNA polymerases are homologs of the β′ and β subunits of *E. coli* RNA polymerase, respectively, and of the equivalent largest subunits of eukaryotic RNA polymerases I, II and III. These subunits interact to form the entry and exit channels for the DNA template, the catalytic center for RNA polymerization and the exit channel for the RNA transcript [1]. The largest and second-largest subunits of RNA polymerases IV and V (abbreviated Pol IV and Pol V) were initially identified upon analysis of the *A. thaliana* genome sequence, which led to the identification of two genes for an atypical fourth class of largest subunit and two genes for an atypical fourth class of second-largest subunit in addition to the canonical Pol I, II and III subunits [2], [3]. Phylogenetic analyses suggest that the atypical subunits arose from duplicated Pol II subunit genes in a multi-step process that began in green algae prior to the evolution of land plants [4] more than 500 million years ago.

For purposes of subunit nomenclature, nuclear RNA polymerases I, II and III in Arabidopsis are designated NRPA, NRPB and NRPC and their largest subunits are NRPA1, NRPB1 and NRPC1. Extending this convention to the atypical polymerases, their largest subunits have been designated either NRPD1a and NRPD1b [5], [6] or RPD1 and RPE1[4]. The latter nomenclature has been adopted, in modified form [7], to allow the naming of Pol IV-specific subunits using the NRPD prefix and the naming of Pol V-specific subunits (formerly Pol IVb) using the NRPE prefix. There are two atypical second-largest polymerase subunit genes, but only one is functional in Arabidopsis and is used by both Pol IV and Pol V, as shown by co-immunoprecipitation, colocalization [8] and genetic evidence [9], [10]. This second-largest subunit gene has the synonymous names NRPD2a (NRPD2 for simplicity) and NRPE2.

The *NRPD1 (NRPD1a)*, *NRPE1 (NRPD1b)* and *NRPD2/NRPE2* genes are not essential for viability [5], [6], [9], [10], unlike the genes encoding the equivalent subunits of Pol I, II and III [5], [11]. However, Pol IV and Pol V subunits localize within the nucleus [5], [8], [12] and are required for the silencing of transgenes, retrotransposons and other endogenous repeats via a 24 nt siRNA-dependent DNA methylation pathway [13]. Pol IV appears to act at the beginning of the RNA-directed DNA methylation pathway because Pol IV colocalizes with endogenous repeat loci that give rise to abundant 24 nt siRNAs and because mutation of Pol IV catalytic subunits causes the loss of 24 nt siRNAs and the mislocalization of other proteins in the pathway [8]. RNA-DEPENDENT RNA POLYMERASE 2 (RDR2) acts downstream of Pol IV, presumably using single-stranded Pol IV transcripts as templates for the production of complementary RNAs. Resulting double-stranded RNAs (dsRNA) are then thought to serve as substrates for DICER-LIKE 3 (DCL3), an RNase III-like endonuclease that cleaves the dsRNAs into 24 nt siRNA duplexes, one strand of which associates with ARGONAUTE 4 (AGO4) to form an RNA-induced silencing complex (RISC). AGO4-RISC presumably uses each siRNA as a guide, targeting cytosine methylation to DNA sequences complementary to the siRNA in a process catalyzed by the *de novo* DNA methyltransferase, DRM2 [14]. Pol V is required for the methylation of target sequences, generating RNA transcripts at target loci that are hypothesized to basepair with AGO4-RISC siRNAs and facilitate the recruitment of DRM2 to the adjacent chromatin [7].

In a previous report, we showed that column fractions enriched for Arabidopsis NRPD2/NRPE2, and therefore presumably containing Pol IV and Pol V complexes, lack detectable promoter-independent RNA polymerase activity using sheared template DNA whereas activity was readily detected in fractions enriched for Pol I, II and III [5]. To explain this negative result, it has been proposed that Pol IV and Pol V may require specialized templates, such as methylated DNA or dsRNA, or may even lack transcriptional activity altogether [5], [6], [8], [10], [15], [16]. However, the NRPD1, NRPE1 and NRPD2/NRPE2 subunits possess minimal Metal A and Metal B motifs typical of RNA polymerase active sites. The Metal A and Metal B sites bind magnesium ions that guide free nucleoside triphosphates into the active site for RNA synthesis, stabilize the transition state of the growing RNA chain and participate in transcript cleavage events during polymerase backtracking, a process which helps prevent polymerase arrest at pause sites [17], [18]. The Metal A site within the largest subunit of multi-subunit RNA polymerases permanently binds a magnesium ion and is formed by three invariant aspartate residues within a nearly invariant NADFDGD motif [19]. The Metal B site is formed by an invariant glutamate and aspartate pair in the second-largest subunit that, in cooperation with one of the aspartates of the Metal A site, transiently binds a second magnesium ion [19]. Mutation of the amino acids that comprise the Metal A or Metal B sites is sufficient to abrogate transcriptional activity in bacteria [20], archaea [21] and eukaryotes [22].

We hypothesized that if RNA Polymerases IV and V function as RNA polymerases, their Metal A and Metal B consensus sequences should be essential for their known biological activities. To test this hypothesis, we conducted site-directed mutagenesis of the Metal A and Metal B motifs within the NRPD1, NRPE1 and NRPD2/NRPE2 subunits, stably incorporated the engineered genes into transgenic plants that were defective for the corresponding endogenous genes and tested for the restoration of Pol IV and Pol V functions *in vivo*. We show that the Metal A and Metal B sites are required for the biological functions of Pol IV and Pol V including siRNA production, RNA-directed DNA methylation and transposon silencing. Additionally, the active sites are required for the distinctive punctate nuclear localization patterns observed for Pol IV and Pol V [5], [8], suggesting that these foci represent Pol IV and Pol V transcription factories [23].

## Results

### Pol IV catalytic subunits retain core sequences of polymerase active sites

**Figure 1:**
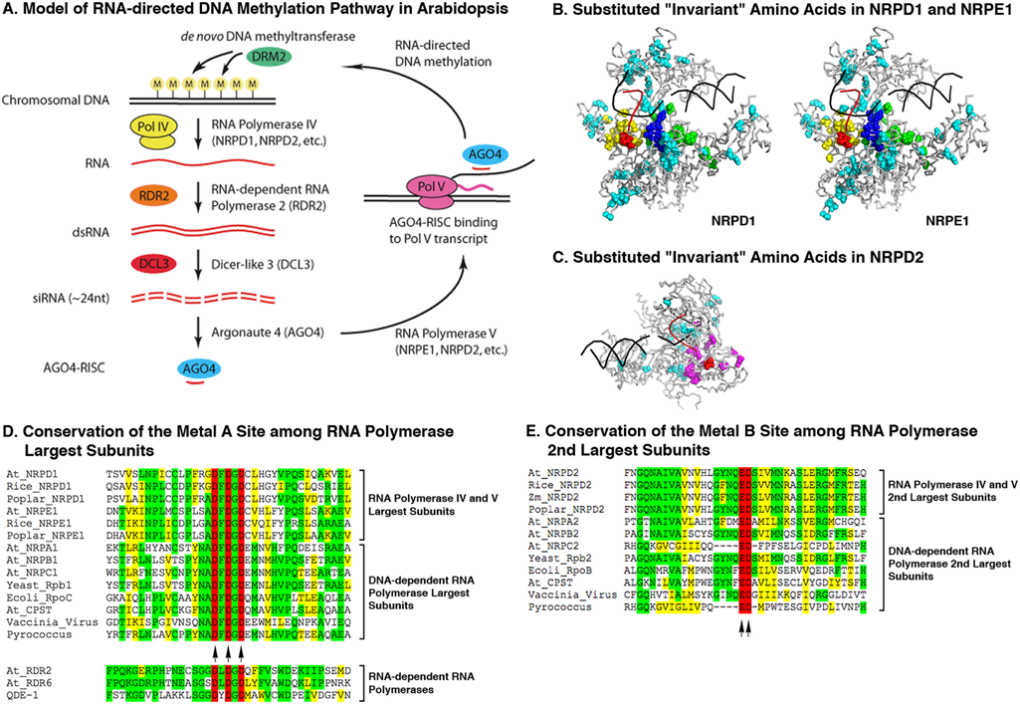
Catalytic residues that comprise the Metal A and Metal B binding sites of DNA-dependent RNA polymerases are conserved in the NRPD1, NRPE1/NRPD1b and NRPD2 subunits. A) Model for the RNA-directed DNA methylation pathway in Arabidopsis. B and C) Positions of NRPD1, NRPE1 and NRPD2 divergence at sites that are invariant in canonical RNA polymerases. The image shows the yeast Pol II Rpb1 and Rbp2 subunits (gray) in complex with the dsDNA substrate (black) and RNA product (red) within Protein Data Bank crystal structure 1R9T (Kornberg laboratory). Amino acids that are invariant among the Arabidopsis Pol I, II and III subunits and yeast Rpb1 or Rpb2, but that are different in NRPD1, NRPE1 or NRPD2, are displayed as spheres. Red spheres highlight the positions of the invariant Metal A and Metal B sites in the largest and second-largest subunits, respectively. Substituted amino acids in the cleft, bridge helix, and active site domains of the largest subunit are colored green, blue and yellow, respectively. Substituted amino acids in the hybrid binding domain of the second-largest subunit are colored magenta. Substituted amino acids in the largest and second-largest subunits that are located outside of these domains are colored cyan. For a complete listing of the highlighted amino acids refer to Table S2. D and E) Multiple protein sequence alignments of RNA polymerase largest and second-largest subunit active site regions. Amino acids highlighted in red and designated by arrows represent the invariant Metal A and Metal B sites. Identical amino acids are highlighted in green and similar amino acids are highlighted in yellow.

Pol IV and Pol V are rapidly-evolving enzymes, with Arabidopsis NRPD1 (formerly NRPD1a) and NRPE1 (formerly NRPD1b) having amino acid substitution rates 20 times greater than the NRPB1 subunit of Pol II, and NRPD2/NRPE2 having a substitution rate 10 times greater than the Pol II NRPB2 subunit [4]. Based on multiple sequence alignments, we identified the amino acid positions that are invariant among Arabidopsis Pol I, II and III and *S. cerevisiae* Pol II, implying that these amino acids are critically important for polymerase structure and function. Interestingly, numerous amino acids that are invariant among the canonical polymerases (i.e. Pol I, II and III) are substituted by other amino acids in Pol IV and Pol V {Herr, 2005 #2694}{Onodera, 2005 #2695}. In Figures 1B and C we mapped the positions of amino acids that are invariant among the canonical polymerases but different in NRPD1, NRPE1 or NRPD2/NRPE2 onto the *S. cerevisiae* Rpb1 and Rpb2 subunit structures in the context of a yeast Pol II elongation complex crystal structure. Interestingly, a large proportion of the “invariant” amino acids that have been substituted in NRPD1, NRPE1 (NRPD1b) and NRPD2/NRPE2 cluster in the vicinity of the catalytic center. In particular, sequences surrounding the Metal A binding site, bridge helix, cleft and funnel domains of NRPD1 and NRPE1 and the hybrid binding region of NRPD2 [1], [19], [24] are hotspots of Pol IV divergence relative to the invariant amino acids of the canonical polymerases (see also Figure S1 and Table S2). These regions govern interactions with the DNA template and the RNA/DNA hybrid that forms between the template and nascent transcript [25].

Multiple sequence alignment in the vicinity of the Metal A and Metal B sites of RNA polymerase largest and second-largest subunits illustrates the sequence divergence that has occurred in Pol IV and Pol V subunits relative to other RNA polymerases (Figures 1D and E). Immediately surrounding the Metal A site in the largest subunit, the sequence NADFDGD is invariant among *E. coli*, chloroplast, archaeal (Pyrococcus), viral, and eukaryotic Pol I, II and III polymerases. This sequence motif is part of an extended sequence, YNADFDGDEMN that is conserved in eukaryotic Pol I, II and III and archaeal polymerases. However, despite having apparently evolved from a duplicated Pol II largest subunit, the NRPD1 subunit of Pol IV has only the core DFDGD sequence that includes the three magnesium-coordinating aspartates. In the NRPE1 (NRPD1b) subunit of Pol V, this core sequence consensus is extended by only one amino acid: the alanine preceding the first aspartate (ADFDGD). Importantly, the consensus sequence DxDGD occurs at the active sites of single-subunit RNA-dependent RNA polymerases, such as Arabidopsis RDR2 and RDR6 or Neurospora QDE-1. Therefore, the conservation of the minimal DFDGD sequence in NRPD1 and NRPE1 is consistent with the hypothesis that these subunits have minimal Metal A sites. Likewise, the NRPD2 subunit utilized by both Pol IV and Pol V contains the core ED motif of the Metal B site as part of an extended G(Y/F)NQEDS motif also present in the second-largest subunit of Pol II. Collectively, these observations suggest that Pol IV and Pol V have Metal A and Metal B sites at their presumptive active sites.

### Pol IV and Pol V Metal A and Metal B motifs are required for siRNA accumulation

**Figure 2:**
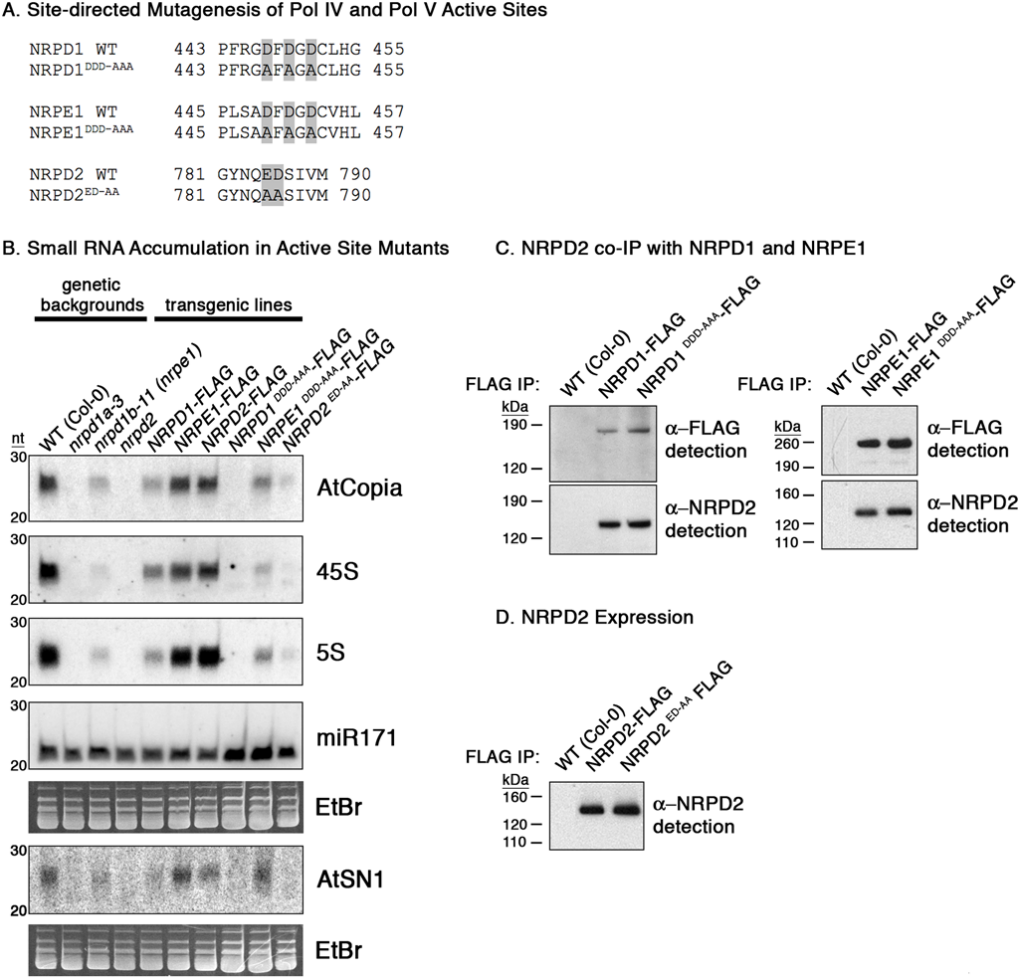
Pol IV and Pol V active site amino acids are required for rescue of small RNA production but not Pol IV or Pol V subunit assembly. A) Acidic amino acids of the Metal A and Metal B sites were mutated to alanines by site-directed mutagenesis. Resulting full-length genomic transgenes were transformed into Arabidopsis *nrpd1a-3*, *nrpd1b-11 (nrpe1)* and *nrpd2a/2b (nrpd2)* homozygous mutants, respectively, as were wild-type versions of each genomic construct. B) RNA blot analysis of small RNAs purified from Arabidopsis inflorescence. Membranes were sequentially probed with body-labeled RNA probes specific for *AtCopia*, 45S rRNA gene intergenic spacer, 5S rRNA gene intergenic spacer, miR171 or *AtSN1* small RNAs. Images of ethidium-bromide stained gels are displayed below the relevant autoradiograms to show that equal amounts of RNA were loaded in each lane. Migration of the 20-nt and 30-nt RNA markers is indicated at the left of each autoradiogram. C and D) Pol IV and Pol V largest subunits bearing active site mutations are indistinguishable from wild-type versions of the proteins in terms of expression level or ability to assemble with the NRPD2 subunit. FLAG-tagged recombinant proteins immunoprecipitated from total protein extracts using anti-FLAG antibodies were detected on immunoblots using FLAG M2 antibody. Membranes were then stripped and re-probed using a polyclonal antibody specific for NRPD2.

**Figure 3:**
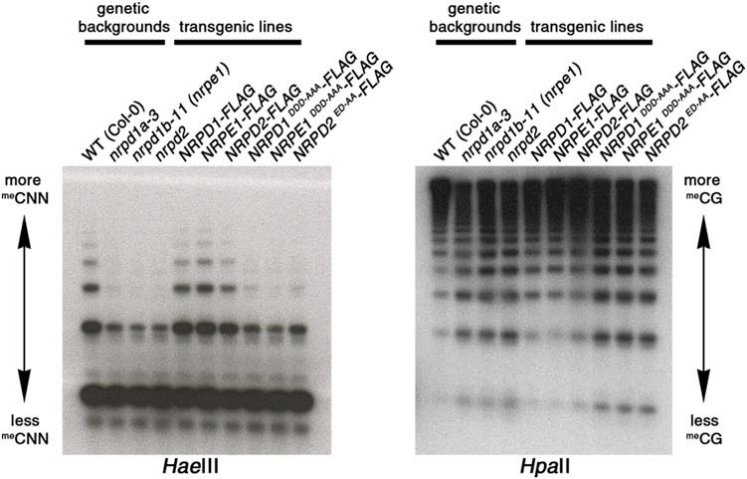
Pol IV and Pol V active site amino acids are required for the RNA-directed methylation of 5S rRNA gene repeats. Southern blot comparison of *Hae*III or *Hpa*II-digested genomic DNA of wild-type (WT), *nrpd1a*, *nrpe1/nrpd1b*, and *nrpd2* mutants or of transgenic lines generated by transforming these mutants with *NRPD1*, *NRPE1/NRPD1b* or *NRPD2a* full-length transgenes whose sequences are either wild-type or are mutated at the Metal A or Metal B sites. Both wild-type and mutant recombinant proteins have FLAG epitope tags at their carboxyl termini.

**Figure 4:**
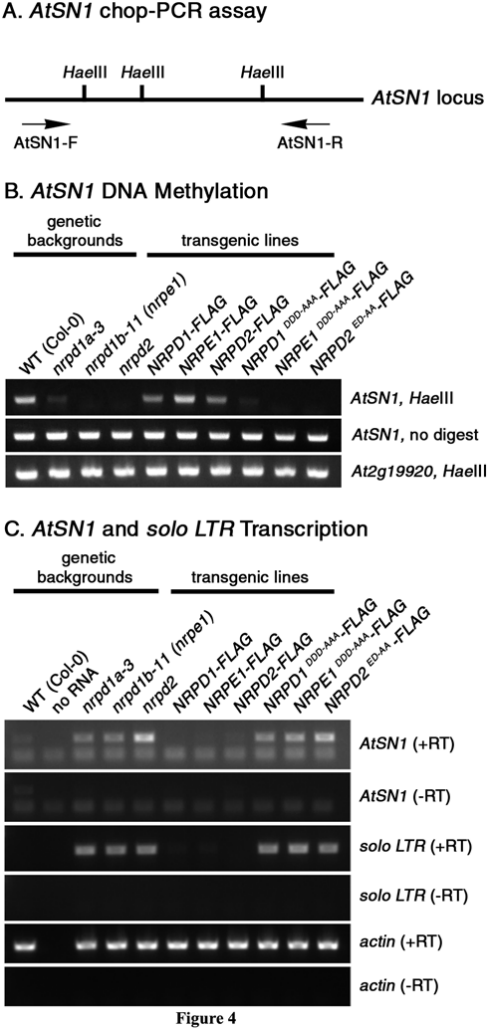
DNA methylation and transcriptional silencing of *AtSN1* retrotransposons requires the Pol IV and Pol V active sites. A) Schematic of an *AtSN1* retroelement locus showing the locations of *Hae*III restriction enzyme sites and flanking PCR primers. B) *AtSN1* DNA methylation analysis using the chop-PCR assay. *AtSN1* loci were PCR amplified from *Hae*III digested or undigested genomic DNA and samples were then subjected to agarose gel electrophoresis and staining with ethidium bromide. Locus *At2g19920* lacks *Hae*III restriction sites and was used as a control. C) RT-PCR analysis of retrotransposon transcription. Random-primed cDNA was used as the template for PCR amplification of *AtSN1* and solo-LTR transcripts. Reactions were then subjected to agarose gel electrophoresis and staining with ethidium bromide. For each genotype, reactions from which reverse transcriptase was omitted (-RT) or for which actin RNA was PCR-amplified serve as controls.

**Figure 5:**
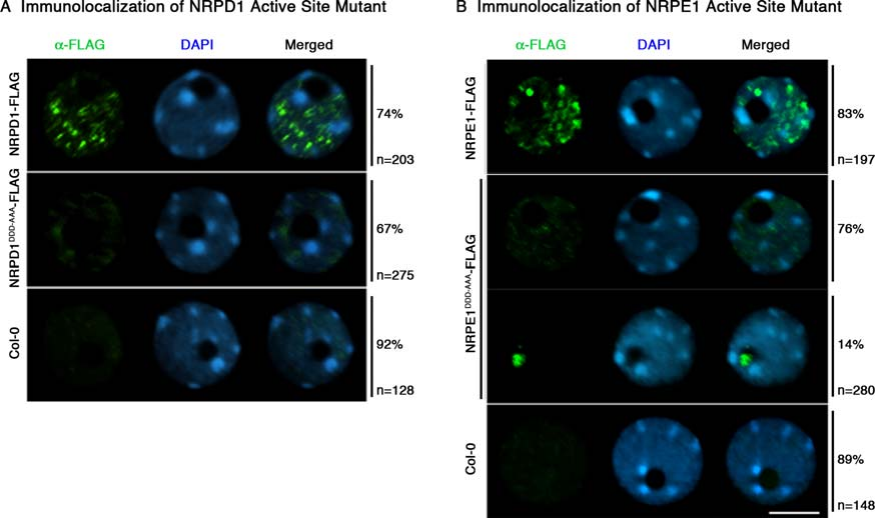
NRPD1 and NRPE1/NRPD1b proteins mutated at their active sites fail to display characteristic Pol IV and Pol V punctate localization patterns in Arabidopsis nuclei. FLAG epitope-tagged NRPD1 and NRPD1^DDD-AAA^ (panel A) or NRPE1 and NRPE1^DDD-AAA^ (panel B) recombinant proteins were immunolocalized (green signal) using anti-FLAG M2 antibody. Nuclei were counterstained with DAPI (blue signal). The percentage of nuclei showing a given localization pattern and the number of nuclei (n) analyzed are indicated to the right of each panel.

To address whether the presumptive active sites of Pol IV and Pol V are required for their functions, we performed site-directed mutagenesis to change the acidic residues of the Metal A and Metal B sites to alanines. Three amino acid substitutions were performed in the largest subunits of Pol IV and Pol V: for NRPD1 these were D447A, D449A and D451A and for NRPE1 (NRPD1b) they were D449A, D451A and D453A. For NRPD2/NRPE2, E785A and D786A mutations were introduced (Figure 2A). Full-length genomic clones bearing these mutations, expressed using the endogenous promoters and containing their complete intron-exon structures, were fused at the C-terminus to a FLAG peptide epitope tag, as were equivalent wild-type (non-mutant) constructs. Resulting *NRPD1* transgenes were introduced into the *nrpd1a-3* null mutant, *NRPE1 (NRPD1b)* transgenes were introduced into the *nrpd1b-11* null mutant and *NRPD2* transgenes were introduced into the *nrpd2a-2 nrpd2b-1* double mutant. Note that the *NRPD2b* gene is a pseudogene due to a frameshift mutation, such that the double mutant is used only as a precaution. The double mutant is hereafter referred to simply as *nrpd2*. Six or more independent transformants for each transgene construct were analyzed to determine the ability of the transgenes to genetically rescue their respective null mutants and all lines for a given construct were found to display the same phenotypes. The active site mutant transgenic lines are abbreviated as *NRPD1^DDD-AAA^-FLAG*, *NRPE1^DDD-AAA^-FLAG* or *NRPD2^ED-AA^-FLAG* in Figures 2, 3, 4, 5.

The requirement for the presumptive Pol IV and Pol V active sites was first tested by comparing the abilities of wild-type or mutant transgenes to rescue the accumulation of siRNAs corresponding to 45S or 5S rRNA gene repeats or *AtCopia* or *AtSN1* retrotransposons (Figure 2B). siRNAs corresponding to these repetitive sequences are predominantly 24 nt in size and are readily detectable in wild-type (WT; ecotype Col-0) plants. However, the siRNAs are eliminated in *nrpd1* or *nrpd2* mutants and are substantially reduced in *nrpe1* (*nrpd1b*) mutants, in agreement with prior studies [6], [8], [9], [10]. In transgenic lines expressing wild-type *NRPD1-FLAG*, *NRPE1-FLAG* or *NRPD2-FLAG* transgenes in their respective mutant backgrounds, siRNA production is restored, albeit to lower than wild-type levels in the case of the *NRPD1* transgene. A delay in flowering time observed in the *nrpd1* mutant, and other mutants affecting the siRNA-directed DNA methylation pathway, is also not fully restored by the NRPD1 transgene (Figure S2), suggesting a correlation between siRNA levels and more rapid flowering. Importantly, no rescue of siRNA levels is observed in transgenic lines expressing the *NRPD1^DDD-AAA^-FLAG* or *NRPE1^DDD-AAA^-FLAG* transgenes; in these lines, siRNA levels are the same as in the *nrpd1a-3* or *nrpd1b-11* mutant parental lines. These results indicate that the Metal A sites of Pol IV (NRPD1) and Pol V (NRPE1/NRPD1b) largest subunits are required for small RNA biogenesis or accumulation. Trace siRNA signals were detected in the *NRPD2^ED-AA^-FLAG* transgenic plants but not in the *nrpd2* mutant parental line (Figure 2B). This suggests that the NRPD2 contribution to the Metal B site is not absolutely required for siRNA biogenesis, but is clearly important.

Two trivial explanations for the results of Figure 2B could be that the Pol IV and Pol V active site mutant proteins are not expressed at levels comparable to their wild-type counterparts or that mutation of the active site region disrupts Pol IV or Pol V subunit assembly. To test these possibilities, anti-FLAG co-immunoprecipitation (co-IP) experiments were performed using equal amounts of total protein extracted from transgenic plants expressing either the wild-type or active site mutant versions of the *NRPD1-FLAG* and *NRPE1-FLAG* transgenes (Figure 2C). Equivalent amounts of the wild-type or mutant large subunits were immunoprecipitated, indicating that they are expressed at similar levels. Moreover, equivalent amounts of the NRPD2/NRPE2 subunit were co-immunoprecipitated by the wild-type or mutated versions of the Pol IV or Pol V largest subunits, suggesting that mutation of the largest subunit active sites does not affect assembly with other subunits. Likewise, the wild-type and active site mutant versions of the *NRPD2-FLAG* transgenes were expressed at similar levels (Figure 2D).

### Pol IV and Pol V active site requirements for DNA methylation

The requirement for the presumptive Pol IV and Pol V active sites in RNA-directed DNA methylation at 5S rRNA gene repeats was tested by Southern blot analysis using the methylation sensitive restriction endonucleases, *Hae*III and *Hpa*II (Figure 3). In this assay, *Hae*III reports on cytosine methylation in CNN motifs whereas *Hpa*II reports on CG methylation. The 5S genes are organized in tandem repeat such that ladders of bands are observed following digestion with methylation-sensitive restriction endonucleases and Southern blot hybridization. Larger bands reflect a relatively high degree of methylation and smaller bands reflect reduced methylation and therefore increased susceptibility to digestion by the enzymes. In *nrpd1*, *nrpe1* (*nrpd1b*) and *nrpd2/nrpe2* mutants, similar losses of CNN or CG methylation occur relative to wild-type (WT) controls. In these mutant backgrounds, methylation is restored to wild-type levels by the corresponding wild-type transgenes (*NRPD1-FLAG*, *NRPE1-FLAG* or *NRPD2-FLAG*, respectively). However, the equivalent transgenes bearing the active site mutations (*NRPD1^DDD-AAA^-FLAG*, *NRPE1^DDD-AAA^-FLAG* and *NRPD2^ED-AA^-FLAG*) fail to rescue the defects in DNA methylation caused by the *nrpd1a-3*, *nrpd1b-11 (nrpe1)* and *nrpd2/nrpe2* mutations.

Like 5S rRNA gene loci, *AtSN1* retrotransposons are subjected to siRNA-directed DNA methylation in a Pol IV and Pol V-dependent manner [6], [9], [10]. We tested *AtSN1* methylation using chop-PCR (Figure 4A). In this assay, genomic DNA is digested (chopped) with *Hae*III and PCR primers flanking the three *Hae*III restriction enzyme sites are then used to amplify the intervening region. If any of the three sites are unmethylated, *Hae*III cuts the template and PCR amplification fails. Only if all three *Hae*III sites are methylated does PCR amplification occur. In wild-type (Col-0) plants, *AtSN1* elements are methylated, rendering them resistant to *Hae*III digestion (Figure 4B). However, in the *nrpd1a-3*, *nrpd1b-11 (nrpe1)* or *nrpd2/nrpe2* mutants, methylation is lost, resulting in *Hae*III susceptibility and the loss of PCR product. Whereas wild-type *NRPD1-FLAG*, *NRPE1-FLAG* and *NRPD2-FLAG* transgenes rescue their respective null mutants and restore DNA methylation at the *AtSN1* loci, the corresponding active site mutants fail to do so (Figure 4B). We conclude that the active sites of NRPD1, NRPE1 and NRPD2/NRPE2 are required for RNA-directed DNA methylation.

### Pol IV and Pol V active site requirements for transcriptional silencing

Consistent with the losses in *AtSN1* siRNA accumulation (Figure 2B) and DNA methylation at *AtSN1* retrotransposons (Figure 4B), silencing of *AtSN1* elements and a retrotransposon-derived solo LTR element [26] are lost in Pol IV and Pol V mutants (Figure 4C). *AtSN1* and solo LTR transcripts are not detected by RT-PCR in wild-type (WT) plants but are apparent in *nrpd1a-3*, *nrpd1b-11* (*nrpe1*) or *nrpd2* mutants. Transforming these mutants with the *NRPD1-FLAG*, *NRPE1-FLAG* or *NRPD2-FLAG* transgenes, respectively, restores *AtSN1* and solo LTR silencing. However, the active site mutant versions of the transgenes fail to restore *AtSN1* or solo LTR silencing in the mutant backgrounds.

### The NRPD1, NRPE1 and NRPD2 active sites are required for the distinctive localization patterns of Pol IV and Pol V

Although NRPD1, NRPE1 and NRPD2/NRPE2 proteins mutated at their presumptive active sites lack detectable *in vivo* function, as shown by their failure to genetically rescue their corresponding null mutants, the proteins are expressed at the same levels as their wild-type counterparts and the mutated largest subunits assemble with the NRPD2/NRPE2 subunit, as shown by co-immunoprecipitation and immunoblotting (Figures 2C, D). Therefore, we investigated the nuclear localization patterns of the proteins mutated at the Metal A and Metal B sites relative to the wild-type proteins (Figure 5). As reported previously [5], [8], [12], immunolocalization of non-mutant NRPD1 and NRPE1 FLAG-tagged proteins reveals that the proteins are localized within punctate foci dispersed throughout the nucleoplasm, with NRPE1 also being found in a “nucleolar dot” [8] that we have interpreted to be a center for siRNA-processing and RISC assembly [8], [12]. Interestingly, the NRPD1 and NRPE1 proteins mutated at their Metal A sites fail to display the distinctive nucleoplasmic puncta or foci. Instead, weak and highly dispersed signals are detected throughout the nucleoplasm. A nucleolar dot signal is observed in 14% of nuclei expressing the NRPE1^DDD-AAA^-FLAG protein despite the lack of detectable nucleoplasmic puncta in these nuclei. Although 83% of wild-type nuclei display an NRPE1 nucleolar dot, these observations suggest that the Metal A site is not required for NRPE1 to associate with the putative siRNA processing center.

## Discussion

Although RNA polymerase activity has not yet been demonstrated *in vitro* for Pol IV or Pol V, our results show that their predicted Metal A and Metal B sites, which are essential for multi-subunit RNA polymerase activity, are required for Pol IV and Pol V biological functions *in vivo*. These results suggest that both Pol IV and Pol V are catalytically active as RNA polymerases. Supporting evidence is that low-level intergenic transcripts that are dependent on Pol V can be detected *in vivo* by using RT-PCR; Pol V physically associates with these loci and production of the intergenic RNAs is abolished in the NRPE1 Metal A site mutant lines we developed in the current study [7]. Although we tested NRPD1 or NRPE1 subunits mutated at all three aspartates of their Metal A sites, genetic evidence suggests that mutation of even one of these aspartates is sufficient to disrupt Pol V function. Specifically, one of nine mutant alleles of NRPE (NRPD1b) identified by Kanno et al. in a screen for mutants disrupting silencing due to RNA-directed DNA methylation [10] results from a single amino acid substitution in the Metal A site (allele *drd3-3*: D451N).

In the vicinity of the Pol IV and Pol V active sites, numerous amino acids that are invariant in Pol I, II and III are missing or replaced by other amino acids. Many of these amino acids occur in regions that influence the predicted template channel, including the bridge helix of the largest subunit, a highly conserved structure from bacterial to eukaryotic polymerases over which the template strand passes en route to the active site [19], [27], [28]. The bridge helices of Arabidopsis Pol I, II and III are approximately 75% identical overall, yet more than half of their invariant amino acids are replaced in NRPD1 and NRPE1 (see Figure S1 and Table S1) [24]. Such alterations in the vicinity of the template channel and active site may facilitate the use of non-conventional templates, including the possible transcription of double-stranded RNA (dsRNA) templates rather than DNA templates. Pol IV is required in several small RNA pathways in which dsRNAs are apparently produced independent of Pol IV action, including a pathway in which siRNA production is triggered by the overlap of RNA transcripts from convergently-transcribed genes [29]. Therefore, transcription of dsRNA by Pol IV is a distinct possibility [3], [24]. Moreover, there is precedent for multi-subunit DNA-dependent RNA polymerases transcribing RNA, including the replication of Hepatitis Delta Virus (HDV) or plant viroid RNAs by Pol II transcription [30], [31]. Yeast Pol II has also been demonstrated to have RNA-dependent RNA polymerase (RdRP) activity although it synthesizes RNA transcripts more slowly than when transcribing DNA and is less processive [32]. It is plausible that the amino acid sequence changes in Pol IV and Pol V largest subunits at sites that are invariant in Pol I, II or III may improve catalytic activity or processivity on alternative templates, such as RNA.

Accumulation of 24 nt siRNAs requires the Metal A consensus sequences of NRPD1 and NRPE1 (Figure 2B). Interestingly, trace amounts of siRNAs are restored in *nrpd2* null mutants transformed with the NRPD2 active site mutant. One explanation for this observation may be that the second-largest subunit's contribution to magnesium ion binding at the Metal B site is slightly less critical than the magnesium binding coordinated by the largest subunit. Consistent with this interpretation, single amino acid substitutions in the Metal B site of an archaeal RNA polymerase were shown to substantially decrease, but not completely abrogate, transcriptional activity [21]. However, the trace amounts of siRNA production that are detected in *NRPD2^ED-AA^-FLAG* lines are apparently not sufficient for rescue of RNA-directed DNA methylation at 5S rRNA genes or *AtSN1* retroelements or for restoration of AtSN1 or solo LTR silencing.

It is noteworthy that the non-mutant *NRPD1-FLAG* transgene did not fully rescue delayed flowering time in the *nrpd1a-3* mutant background to that of wild-type plants (see Supplemental data), nor did the transgene fully rescue siRNA levels (see Figure 2B). Nonetheless, *5S* rRNA gene and *AtSN1* DNA methylation levels were fully rescued by the *NRPD1-FLAG* transgene. Collectively, these observations suggest that tissue-specific differences in transgene expression, or different siRNA level thresholds, may explain the different degrees of transgene effectiveness in the various assays.

Despite evidence that NRPD1 and NRPE1/NRPD1b active site mutants are expressed at the same levels as non-mutant recombinant proteins and are not impaired in their ability to assemble with the NRPD2 subunit, the active site mutants fail to display the characteristic punctate nucleoplasmic localization patterns typical of wild-type NRPD1 or NRPE1. One possibility could be that active site mutants are unable to bind their template(s) and thus never localized to chromatin. Although we cannot rule out this possibility, *E. coli* RNA polymerase that is mutated at the Metal A site is still able to bind DNA and form an open-promoter complex, despite being transcriptionally inactive [20]. Therefore, it is plausible that Pol IV or Pol V complexes bearing active site mutations can bind and occupy their templates. Individual loci bound by single Pol IV or Pol V molecules would likely escape detection in our immunolocalization assays. Therefore, we think it most likely that the nucleoplasmic foci at which Pol IV and Pol V are concentrated in wild-type nuclei represent transcription factories in which Pol IV or Pol V-transcribed sequences coalesce, analogous to the transcription factories observed for *E. coli* RNA polymerase or eukaryotic RNA Polymerases I, II or III [23]. If so, heterochromatic regions that are subject to Pol IV or Pol V-dependent chromatin modifications may coalesce as a result of Pol IV or Pol V transcription.

## Methods

### Mutant plant strains

*Arabidopsis thaliana* mutants *nrpd1a-3*, *nrpd2a-2 nrpd2b-1* (abbreviated as *nrpd2a/2b*) and *nrpd1b-11* were described previously [5], [8]. All are apparent null mutants resulting from *Agrobacterium tumefaciens*-mediated, multi-kb insertions that disrupt the genes [33].

### Multiple sequence alignment

GenBank sequences for largest and second-largest RNA polymerase subunit alignments were those described previously (see supplemental material of reference [5]), with the addition of *Zea mays* NRPD2 (AAY45706), Arabidopsis RDR2 (NP_192851), Arabidopsis RDR6 (NP_190519) and *Neurospora crassa* QDE-1 (CAB42634). NRPD1 (LG_I, 8313188-8324531), NRPE1 (LG_III, 17406212-17419838) and NRPD2 (LG_XVIII, 6286719-6297405) sequences from poplar were identified using the *Poplulus trichocarpa* unmasked genome assembly v1.1 by JGI and the tBLASTn tool with Arabidopsis protein queries. Sequences were aligned using ClustalW2 and colored using BOXSHADE.

### Site-directed mutagenesis

Site-directed ligase independent mutagenesis (SLIM) [34] was performed to change aspartates to alanines at the Metal A sites of *Arabidopsis* NRPD1 (NRPD1a) (D447A, D449A, D451A) and NRPE1 (NRPD1b) (D449A, D451A, D453A) and to mutate the Metal B site of NRPD2a (E785A, D786A). Nucleotides 910-2232 of the NRPD1a genomic sequence were PCR amplified from pENTR-NRPD1a with NRPD1a active site-F and NRPD1a active site-R primers (see Table S1 for primer sequences) and Pfu Ultra (Stratagene). The resulting PCR product was cloned into the pCR4-TOPO vector (Invitrogen) for subsequent mutation using primers NRPD1a DDD/AAA-F, NRPD1a mut-F, NRPD1a DDD/AAA-R and NRPD1a mut-R (see Table S1). The resulting mutated sequence within plasmid pCR4-NRPD1a^DDD-AAA^ was then subcloned back into the pENTR-NRPD1a genomic clone by digesting pENTR-NRPD1a and the pCR4-NRPD1a^DDD-AAA^ active site region PCR clone with *Sac*I, gel purifying the desired fragments and performing a standard ligation reaction. The pENTR-NRPD1b (NRPE1) genomic clone was mutated with primers NRPD1b DDD/AAA-F, NRPD1b mut-F, NRPD1b DDD/AAA-R and NRPD1b mut-R (see Table S1). The pDONR-NRPD2a genomic clone was mutated with primers NRPD2a ED/AA-F, NRPD2a mut-F, NRPD2a ED/AA-R and NRPD2a mut-R (see Table S1). Proper ligation at cloning junctions and at mutated active sites was confirmed by DNA sequencing.

### Generation of transgenic lines

The cloning of *NRPD1* (*NRPD1a*) and *NRPE1* (*NRPD1b*) genomic sequences and generation of *NRPD1-FLAG* and *NRPE1 (NRPD1b)-FLAG* transgenic lines that rescue the *nrpd1a-3* or *nrpd1b-11* null mutants, respectively was described previously [8]. The full-length *NRPD2a* genomic sequence, including 1310 bp upstream of the translation start site, was amplified by PCR from *A. thaliana* (ecotype Col-0) genomic DNA using NRPD2a BP-F and NRPD2a BP-R primers (see Table S1) and Pfu Ultra (Stratagene), cloned into the pDONR221 vector using BP Clonase (Invitrogen) and confirmed by DNA sequencing. The pDONR-NRPD2a, pENTR-NRPD1^DDD-AAA^, pENTR-NRPE1^DDD-AAA^ and pDONR-NRPD2a^ED-AA^ full-length genomic clones were recombined into pEarleyGate 302 [35] in order to add a C-terminal FLAG epitope tag in lieu of the normal stop codon; LR Clonase (Invitrogen) was used for these recombination reactions. Resulting plasmids were transformed into *Agrobacterium tumefaciens* strain GV3101 and homozygous *nrpd1a*, *nrpe1/nrpd1b* or *nrpd2* mutant plants were transformed with the corresponding transgenes using the floral dip method [36]. Seeds of dipped plants were sown and transformants were selected by spraying seedlings with BASTA herbicide. BASTA-resistant primary transformants (T1 generation plants) were then assayed by Southern blot analysis to test their 5S rRNA gene repeat methylation status. All lines displayed equivalent levels of rescue, in the case of wild-type transgenes, or lack of rescue in the case of mutant transgenes (Figure S1). T2 generation transgenic plants were used for all experiments depicted in the figures, unless indicated otherwise.

### Small RNA blot hybridization

RNA was isolated from 300 mg of inflorescence tissue using the mirVana miRNA isolation kit (Ambion). RNA samples (9.5 µg each) were resolved by gel electrophoresis, transferred to nylon membrane and hybridized to radioactive probes as described previously [5]. The *AtSN1* RNA probe, body-labeled with α^32^P-CTP, was prepared according to [37]. *AtCopia*, 45S rRNA gene and 5S rRNA (siR1003) probes were prepared according to [8]. The miR171 riboprobe was generated using the mirVana probe construction kit (Ambion) in conjunction with DNA oligonucleotide miR171T7: 5′TGATTGAGCCGCGCCAATATCcctgtctc3′.

### DNA methylation assays

Southern blot analysis was performed using 250 ng of *Hae*III or *Hpa*II-digested genomic DNA isolated from leaves of 3 to 4-week old plants. Digested DNA was subjected to agarose gel electrophoresis and transferred to uncharged nylon membranes. The 5S rRNA gene probe, labeled with a^32^P-dCTP, was generated by random priming of a full-length 5S gene repeat amplified by PCR from clone pCT4.2 [38]. Probe hybridization and autoradiography were according to standard methods [39]. The *AtSN1* DNA methylation assay involving PCR amplification of undigested or *Hae*III-digested genomic DNA was performed as described previously [6].

### RT-PCR

RNA (∼1 µg) isolated from 3 to 4-week old leaf tissue was treated with RQ1 DNase (Promega) and used to generate random-primed cDNA using degenerate dN6 primers (NEB) and Superscript III Reverse Transcriptase (Invitrogen) according to the manufacturer's instructions. *AtSN1* RT-F and *AtSN1* RT-R primers were used to amplify *AtSN1* transcripts from the cDNA with GoTaq Green (Promega) and samples were analyzed by agarose gel electrophoresis.

### Immunoprecipitation and detection of epitope-tagged proteins

Immunoprecipitation and immunoblot detection of Pol IV and Pol Vproteins was performed using 4.0 g of 3-week old leaf tissue from T3 generation plants, as described previously [8]. Immunolocalization of FLAG-tagged proteins was performed using nuclei of 28-day old leaves, as previously described [8].

## Supporting Information

Table S1 DNA oligonucleotides used in this study
(0.07 MB DOC)

Table S2 Positions of amino acids that are invariant among Arabidopsis Pol I, II and III and yeast Pol II but have diverged in Arabidopsis Pol IV and Pol V largest and second-largest subunits. The table lists amino acids, numbered according to the PDB:1R9T crystal structure for yeast Pol II, and the changes at these positions in NRPD1, NRPE1 or NRPD2. These are the amino acids highlighted in Figures 1B and 1C. Amino acid substitutions are based on the multiple alignments shown in Figure S1 for the RNAP largest subunits and in the supplemental material of Onodera et al (2005) for the RNAP second-largest subunits. Major structural features, according to Cramer et al (2001), are designated to the left of the tables.
(0.18 MB DOC)

Figure S1 Multiple alignment of A. thaliana RNAP Largest Subunits and the Yeast Pol II Largest Subunit. Full-length protein sequences for A. thaliana NRPA1 (At3g57660), NRPB1 (At4g35800), NRPC1 (At5g60040), NRPD1 (At1g63020), NRPE1 (At2g40030) and S. cerevisiae Rpb1 were aligned using ClustalW2 (http://www.ebi.ac.uk/Tools/clustalw2/index.html) in conjunction with final editing by hand. Alignments were colored using BOXSHADE v3.21 (http://www.ch.embnet.org/software/BOX_form.html). DNA-dependent RNA polymerase conserved domains A to H are underlined and designated to the right of the alignments. Yeast Pol II structural features, according to Cramer et al (2001), are designated below the alignments. Regions that make contact with other RNAP subunits are designated in italics above the alignments. The Metal A site is designated with asterisks above the alignment.
(0.18 MB DOC)

Figure S2 Flowering time control is dependent upon the Pol IV and Pol V active sites. nrpd1a, nrpe1/nrpd1b and nrpd2 mutants, or transgenic lines generated by transforming these mutants with wild-type or active site mutant versions of NRPD1, NRPE1/NRPD1b or NRPD2a full-length transgenes, were grown side-by-side under short day conditions (8 hours light/16 hours dark). The positions of pots were changed every 4–6 days according to a randomized plot design. The total number of rosette leaves for each plant was counted when the bolt (flower stalk) achieved a height of 5 cm. The histograms show the average number of leaves at flowering+/−the standard error of the mean. Asterisks denote mean values that are significantly different (p<0.05) from the wild-type (WT; ecotype Col-0) control population as determined by using the Student t-Test; a double asterisk denotes a value that is significantly different from both the WT and nrpd1a-3 controls. The number of individual plants analyzed for each genotype is denoted by the numeric value inside each vertical bar. As expected, based on prior studies [1], [2], nrpd1a-3, nrpd1b-11 (nrpe1) and nrpd2 mutant plants were significantly delayed in flowering relative to wild-type plants. Flowering time of the mutants was unaffected by transforming them with the NRPD1, NRPE1 or NRPD2 active site mutant transgenes. However, wild-type flowering time was restored by the non-mutant NRPE1-FLAG or NRPD2-FLAG transgenes. It is noteworthy that the non-mutant NRPD1-FLAG transgene did not fully restore flowering time in the nrpd1a-3 mutant background to that of wild-type plants, perhaps reflecting the incomplete rescue of siRNA levels shown in Figure 2B.
(0.45 MB TIF)
