# Supplementary material for: Metal A and Metal B Sites of Nuclear RNA Polymerases Pol IV and Pol V Are Required for siRNA-Dependent DNA Methylation and Gene Silencing
Source: PLoS One. 2009 Jan 1;4(1):e4110. doi: 10.1371/journal.pone.0004110 (PMC2605557; doi:10.1371/journal.pone.0004110)
Supplement: Table S1 — DNA oligonucleotides used in this study (0.07 MB DOC) [file pone.0004110.s001.doc]

| Target | Primer | Sequence (5’ to 3’) | Application |
| --- | --- | --- | --- |
| At3g23780 | NRPD2a BP-F  NRPD2a BP-R | GGGGACAAGTTTGTACAAAAAAGCAGGCTAAAGATCAGTTCCAAGTTGGTTGGC  GGGGACCACTTTGTACAAGAAAGCTGGGTGCGCATAGCTTGGTGTCGAAGTTGAGAGTG | Clone NRPD2a into pDONR221 |
| At1g63020 | NRPD1a active site-F  NRPD1a active site-R | CACCGGCGAATAATAACGCATGCACAGG  GAATAGCTGCATTCCCGTCCATTG | Amplify the NRPD1a active site region |
| At1g63020 | NRPD1a DDD/AAA-F  NRPD1a mut-F  NRPD1a DDD/AAA-R  NRPD1a mut-R | GGTGCTTTTGCTGGAGCTTGTCTCCACGGTTACGTTCTTCAGTC  CTCCACGGTTACGTTCCTCAGTC  ACAAGCTCCAGCAAAAGCACCACGGAACGGCAAACAGCAGATC  ACGGAACGGCAAACAGCAGATC | Mutagenesis |
| At2g40030 | NRPD1b DDD/AAA-F  NRPD1b mut-F  NRPD1b DDD/AAA-R  NRPD1b mut-R | GCTGCTTTTGCTGGTGCTTGTGTCCATTTGTTCTACCCTCAGTCTCTTAGTG  GTCCATTTGTTCTACCCTCAGTCTCTTAGTG  ACAAGCACCAGCAAAAGCAGCACTGAGGGGGCTACACATCAGAG  ACTGAGGGGGCTACACATCAGAG | Mutagenesis |
| At3g23780 | NRPD2a ED/AA-F  NRPD2a mut -F  NRPD2a ED/AA-R  NRPD2a mut-R | CAACCAAGCGGCTTCCATTGTGATGAACAAGGCTTCATTGGAACGTG  TGATGAACAAGGCTTCATTGGAACGTG  CAATGGAAGCCGCTTGGTTGTACCCGAGATGAACATTCACAGCAAC  TACCCGAGATGAACATTCACAGCAAC | Mutagenesis |
| AtSN1 | AtSN1-F  AtSN1-R | AGGATTTATTTCAATCCACGAACCT  CGACTCCCATAAGTAACGAGTTG | Chop-PCR  (Herr et al., 2005) |
| At2g19920 | AtSN1 control-F  AtSN1 control-R | Ctctgggttacctttcaggaatcag  Ctaaattgaagagcttacctgcttg | Chop-PCR control  (Herr et al., 2005) |
| AtSN1 | AtSN1 RT-F  AtSN1 RT-R | ACCAACGTGCTGTTGGCCCAGTGGTAAATC  AAAATAAGTGGTGGTTGTACAAGC | RT-PCR  (Herr et al., 2005) |
| solo LTR | solo LTR-F  solo LTR-R | ATCAATTATTATGTCATGTTAAAACCGATTG  TGTTTCGAGTTTTATTCTCTCTAGTCTTCATT | RT-PCR  (Wierzbicki unpublished) |
| Actin | Actin-F  Actin-R | TCATACTAGTCTCGAGAGATGACTCAGATCATGTTTGAG  TCATTCTAGAGGCGCGCCACAATTTCCCGTTCTGCGGTAG | RT-PCR  (Herr et al., 2005) |
