# Supplementary material for: Metal A and Metal B Sites of Nuclear RNA Polymerases Pol IV and Pol V Are Required for siRNA-Dependent DNA Methylation and Gene Silencing
Source: PLoS One. 2009 Jan 1;4(1):e4110. doi: 10.1371/journal.pone.0004110 (PMC2605557; doi:10.1371/journal.pone.0004110)
Supplement: Table S2 — Positions of amino acids that are invariant among Arabidopsis Pol I, II and III and yeast Pol II but have diverged in Arabidopsis Pol IV and Pol V largest and second-largest subunits. The table lists amino acids, numbered according to the PDB:1R9T crystal structure for yeast Pol II, and the changes at these positions in NRPD1, NRPE1 or NRPD2. These are the amino acids highlighted in Figures 1B and 1C. Amino acid substitutions are based on the multiple alignments shown in Figure S1 for the RNAP largest subunits and in the supplemental material of Onodera et al (2005) for the RNAP second-largest subunits. Major structural features, according to Cramer et al (2001), are designated to the left of the tables. (0.18 MB DOC) [file pone.0004110.s002.doc]

| ScRpb1:NRPD1  Clamp core | ScRpb1:NRPE1 |  | ScRpb2:NRPD2 |
| --- | --- | --- | --- |
| G52Q | G52Q |  | V44I |
| L53V | - |  | F51Y |
| - | D55N |  | I172V |
| P78E | P78E |  | L174V |
| L86F | - |  | L181T |
| P89S  Clamp  head | - |  | L189N |
| L202Q  Clamp core | - |  | E194D |
| P242T | - |  | I204V |
| - | R247S |  | N221K |
| P248V | P248V |  | I269K |
| E259R | E259S | Fork | D396E |
| T263D | T263R |  | L461E |
| L266T | - | External 1 | L514P |
| N273V | N273V |  | P524F |
| L276E | L276I |  | I743L |
| Q297S | Q297F |  | P745L |
| I325S | I325D |  | A753C |
| L329K | L329S |  | N762D |
| K332G | K332W |  | Q763H |
| G334R | G334E |  | S764G |
| R337K | - |  | P765R |
| - | G342R |  | N767V |
| - | R344G |  | M773H |
| V345S  Active site | V345S |  | K775Q |
| I353V | - |  | M778I |
| - | P357A |  | S844A |
| - | L374I |  | D894E |
| T375Q | - |  | L898F |
| P377S  Dock | P377E | Hybrid binding  Hybrid binding  Protrusion  Lobe  Wall | D951Q |
| V380L | - |  | R983M |
| G395- | G395- |  | D998N |
| P396- | P396- |  | I1011V |
| P400- | P400- |  | M1021Q |
| G401- | G401- |  | Y1091F |
| R412V | R412T |  | V1099S |
| V432I | - |  | G1121D |
| H435S | H435R |  | R1129K |
| -  Active site | L443F |  | G1167K |
| Q447P | Q447P |  | |
| L450I | L450T |  | |
| M456I | M456Q |  | |
| H458M | H458L |  | |
| T467V | - |  | |
| R469S | R469K |  | |
| Y478F | Y478L |  | |
| N479R | N479S |  | |
| A480G | - |  | |
| E486C | E486C |  | |
| M487L | M487V |  | |
| N488H | N488H |  | |
| H490Y | H490F |  | |
| R498K  Pore | R498K |  | |
| D538N | D538R |  | |
| T539C | T539V |  | |
| ScRpb1:NRPD1  Pore | ScRpb1:NRPE1 |  | |
| F540Y | - |  | |
| - | I565L |  | |
| P568A | P568S |  | |
| L571Q | L571A |  | |
| - | G574V |  | |
| K575M | K575F |  | |
| G615F | G615F |  | |
| K619S | K619V |  | |
| L629N | L629I |  | |
| F662L  Funnel | - |  | |
| G665S | G665S |  | |
| G707A | G707- |  | |
| - | L722- |  | |
| N723A | N723- |  | |
| - | N741Y |  | |
| - | M746L |  | |
| - | G750K |  | |
| - | K752N |  | |
| - | G753S |  | |
| S754N | S754A |  | |
| N575K | N757K |  | |
| Q767L | Q767L |  | |
| G772V | G772K |  | |
| R774L | R774K |  | |
| L784C | L784M |  | |
| P785A | P785A |  | |
| F787W | - |  | |
| P794L | P794R |  | |
| F799V | F799I |  | |
| F815V  Bridge helix | F815A |  | |
| M818V | M818I |  | |
| G820S | G820A |  | |
| E822D | - |  | |
| G823S | G823V |  | |
| L824S | L824I |  | |
| D826S | D826R |  | |
| T827G | T827S |  | |
| A828N | A828S |  | |
| V829A | V829R |  | |
| K830D | K830G |  | |
| T831L | T831L |  | |
| Y836T | Y836T |  | |
| -  Cleft | R839K |  | |
| K843F | K843A |  | |
| E846R | E846R |  | |
| V850A | V850I |  | |
| - | Y852N |  | |
| - | G861S |  | |
| G869E | - |  | |
| -  Foot | G872S |  | |
| D874V | D874R |  | |
| L956- | L956- |  | |
| N959- | N959- |  | |
| Q1070C  Cleft | Q1070T |  | |
| S1071A | S1071A |  | |
| G1073S | G1073S |  | |
| ScRpb1:NRPD1 | ScRpb1:NRPE1 |  | |
| -  Cleft domain | E1074N |  | |
| P1075A | - |  | |
| T1077Y | T1077Y |  | |
| Q1078S | Q1078K |  | |
| M1079A | M1079A |  | |
| T1080L | T1080V |  | |
| L1081D | - |  | |
| T1083P | T1083S |  | |
| F1084I | F1084S |  | |
| H1085S | H1085P |  | |
| A1087L | A1087S |  | |
| G1088E | G1088N |  | |
| T1095L | T1095K |  | |
| G1097N | G1097V |  | |
| P1099L | P1099L |  | |
| R1100E | R1100C |  | |
| E1103S | E1103N |  | |
| I1104K | I1104F |  | |
| T1113S | T1113I |  | |
| P1114L | P1114L |  | |
| - | L1120H |  | |
| A1131S | - |  | |
| T1142S  Jaw | T1142S |  | |
| E1151M | E1151L |  | |
| W1191S | W1191I |  | |
| R1199Q  Cleft domain | R1199K |  | |
| V1282I | V1282I |  | |
| L1306V | - |  | |
| - | G1310V |  | |
| N1330D | N1330Y |  | |
| E1342D | E1342S |  | |
| - | A1343C |  | |
| - | R1345F |  | |
| E1351N | E1351R |  | |
| V1355A | V1355S |  | |
| - | G1360S |  | |
| R1366E | R1366E |  | |
| - | D1373N |  | |
| M1375L | - |  | |
| T1376S  Clamp  core | - |  | |
| R1386A | R1386S |  | |
| - | F1402L |  | |
| E1403S | E1403I |  | |
