# Supplementary material for: Metal A and Metal B Sites of Nuclear RNA Polymerases Pol IV and Pol V Are Required for siRNA-Dependent DNA Methylation and Gene Silencing
Source: PLoS One. 2009 Jan 1;4(1):e4110. doi: 10.1371/journal.pone.0004110 (PMC2605557; doi:10.1371/journal.pone.0004110)
Supplement: Figure S1 — Multiple alignment of A. thaliana RNAP Largest Subunits and the Yeast Pol II Largest Subunit. Full-length protein sequences for A. thaliana NRPA1 (At3g57660), NRPB1 (At4g35800), NRPC1 (At5g60040), NRPD1 (At1g63020), NRPE1 (At2g40030) and S. cerevisiae Rpb1 were aligned using ClustalW2 (http://www.ebi.ac.uk/Tools/clustalw2/index.html) in conjunction with final editing by hand. Alignments were colored using BOXSHADE v3.21 (http://www.ch.embnet.org/software/BOX_form.html). DNA-dependent RNA polymerase conserved domains A to H are underlined and designated to the right of the alignments. Yeast Pol II structural features, according to Cramer et al (2001), are designated below the alignments. Regions that make contact with other RNAP subunits are designated in italics above the alignments. The Metal A site is designated with asterisks above the alignment. (0.18 MB DOC) [file pone.0004110.s003.doc]

**Figure S1.** Multiple alignment of *A. thaliana* RNAP Largest Subunits and the Yeast Pol II Largest Subunit. Full-length protein sequences for *A. thaliana* NRPA1 (At3g57660), NRPB1 (At4g35800), NRPC1 (At5g60040), NRPD1 (At1g63020), NRPE1 (At2g40030) and *S. cerevisiae* Rpb1 were aligned using ClustalW2 (<http://www.ebi.ac.uk/Tools/clustalw2/index.html>) in conjunction with final editing by hand. Alignments were colored using BOXSHADE v3.21 (<http://www.ch.embnet.org/software/BOX_form.html>). DNA-dependent RNA polymerase conserved domains A to H are underlined and designated to the right of the alignments. Yeast Pol II structural features, according to Cramer et al (2001), are designated below the alignments. Regions that make contact with other RNAP subunits are designated in italics above the alignments. The Metal A site is designated with asterisks above the alignment.

*Rpb2 Interaction*

NRPD1 1 ------------MEDDCEELQVPVGTLTSIGFSISNNNDRDKMSVLEV------------

NRPE1 1 -------------MEEESTSEILDGEIVGITFALASHHEICIQSISESAI----------

NRPA1 1 MAHAQTTEVCLSFHRSLLFPMGASQVVESVRFSFMTEQDVRKHSFLKVTSPILHDNVGN-

NRPB1 1 ------------MDTRFPFSPAEVSKVRVVQFGILSPDEIRQMSVIHVEHSETTEKGK--

NRPC1 1 ----METKMEIEFTKKPYIEDVGPLKIKSINFSVLSDLEVMKAAEVQVWNIGLYDHSFK-

ScRpb1 1 -------------MVGQQYSSAPLRTVKEVQFGLFSPEEVRAISVAKIRFPETMDETQTR

Clamp core

NRPD1 37 EAPNQVTDSRLGLPNPDSVCRTCGSKDRKVCEGHFGVINFAYSIINPYFLKEVAALLNKI

NRPE1 38 NHPSQLTNAFLGLPLEFGKCESCGATEPDKCEGHFGYIQLPVPIYHPAHVNELKQMLSLL

A

NRPA1 60 PFPGGLYDLKLGPKDDKQACNSCGQL-KLACPGHCGHIELVFPIYHPLLFNLLFNFLQRA

NRPB1 47 PKVGGLSDTRLGTIDRKVKCETCMAN-MAECPGHFGYLELAKPMYHVGFMKTVLSIMRCV

NRPC1 56 PYENGLLDPRMGPPNKKSICTTCEGN-FQNCPGHYGYLKLDLPVYNVGYFNFILDILKCI

ScRpb1 48 AKIGGLNDPRLGSIDRNLKCQTCQEG-MNECPGHFGHIDLAKPVFHVGFIAKIKKVCECV

Clamp core

Clamp head

NRPD1 97 CPGCKYIRKKQFQITEDQPERCRYCT----------LNTGYPLMKFRVTTKEVFRRSG--

NRPE1 98 CLKCLKIKKAKGTSGGLADR---------------------LLGVCCEEASQISIKDR--

NRPA1 119 CFFCHHFMAKPEDVERAVSQLKLIIKGDIVSAKQLESNTPTKSKSSDESCESVVTTDSSE

NRPB1 106 CFNCSKILADEVCRSLFRQAMKIK-------------NPKNRLKKILDACKNKTKCDGGD

NRPC1 115 CKRCSNMLLDEKLYEDHLRKMRNPRM---------EPLKKTELAKAVVKKCSTMASQRII

ScRpb1 107 CMHCGKLLLDEHN-ELMRQALAIK-------------DSKKRFAAIWTLCKTKMVCET--

Clamp head

NRPD1 145 --IVVEVNEESLMKLKKRGVLTLPPDYWSFLP----------------------------

NRPE1 135 -----ASDGASYLELKLPSRSRLQPGCWNFLER---------------------------

NRPA1 179 ECEDSDVEDQRWTSLQFAEVTAVLKNFMRLSSKSCSRCKGINPKLEKPMFGWVRMRAMKD

NRPB1 153 DIDDVQSHSTDEPVKKSRGGCGAQQPKLTIEG----------------------------

NRPC1 166 TCKKCGYLNGMVKKIAAQFGIGISHDRSKIHG----------------------------

ScRpb1 151 ---DVPSE-DDPTQLVSRGGCGNTQPTIRKDG----------------------------

Clamp head

NRPD1 175 -------------------------------------------QDSNIDESCLKPTRRII

NRPE1 163 ---------------------------------------------------YGYRYGSDY

NRPA1 239 SDVGANVIRGLKLKKSTSSVENPDGFDDSGIDALSEVEDGDKETREKSTEVAAEFEEHNS

NRPB1 185 ----------------------------------MKMIAEYKIQRKKNDEPDQLPEPAER

NRPC1 198 ----------------------------------GEIDECKSAISHTKQST---AAINPL

ScRpb1 179 ----------------------------------LKLVGSWKKDRATGDAD----EPELR

Clamp head

NRPD1 192 THAQVYALLLGIDQRLIKKDIP----------------MFNSLGLTSFPVTPNGYRVTEI

NRPE1 172 TRPLLAREVKEILRRIPEESRKKLTAKGHI--------PQEGYILEYLPVPPNCLSVPEA

NRPA1 299 KRDLLPSEVRNILKHLWQNEHEFCSFIGDLWQSGSEKIDYSMFFLESVLVPPTKFRPPTT

NRPB1 211 KQTLGADRVLSVLKRISDADCQLLGFNPKFA-------RPDWMILEVLPIPPPPVRPSVM

NRPC1 221 TYVLDPNLVLGLFKRMSDKDCELL---YIAY-------RPENLIITCMLVPPLSIRPSVM

ScRpb1 201 --VLSTEEILNIFKHISVKDFTSLGFNEVFS-------RPEWMILTCLPVPPPPVRPSIS

B

Clamp head

Clamp core

NRPD1 236 VHQFNGARLI-FDERTRIYKKLVGFEGNTLELSSRVMECMQYSRLFSETVSSSKDSANPY

NRPE1 224 SDGFSTMSVDPSRIELKDVLKKVIAIKSSRSGETNFESHKAEASEMFRVVDTYLQVRGTA

NRPA1 359 GGD-SVMEHP-QTVGLNKVIESNNILGNACTNKLDQSKVIFRWRNLQESVNVLFDSKTAT

NRPB1 264 MDATSRSEDD-LTHQLAMIIRHNENLKRQEKNGAPAHIISEFTQLLQFHIATYFDNELPG

NRPC1 271 IGGIQSNEND-LTARLKQIILGNASLHKILSQPTSSPKNMQVWDTVQIEVARYINSEVRG

B

ScRpb1 252 FNESQRGEDD-LTFKLADILKANISLETLEHNGAPHHAIEEAESLLQFHVATYMDNDIAG

Clamp core

NRPD1 295 ---------------QKKSDTPKLCGLRFMKDVLLGKRSDHTFRTVVVGDPSLKLNEIGI

NRPE1 284 KAARN--IDMRYGVSKISDSSSSKAWTEKMRTLFIRKGSGFSSRSVITGDAYRHVNEVGI

NRPA1 417 ---------VQSQRDSSGICQLLEKKEGLFRQKMMGKRVNHACRSVISPDPYIAVNDIGI

NRPB1 323 ----QPRATQKSGRPIKSICSRLKAKEGRIRGNLMGKRVDFSARTVITPDPTINIDELGV

C

NRPC1 330 ------CQNQPEEHPLSGILQRLKGKGGRFRANLSGKRVEFTGRTVISPDPNLKITEVGI

ScRpb1 311 ----QPQALQKSGRPVKSIRARLKGKEGRIRGNLMGKRVDFSARTVISGDPNLELDQVGV

Clamp core

Active site

NRPD1 340 PESIAKRLQVSEHLNQCNKERLVTSFVP------------------TLLDNKEMHVRRGD

NRPE1 342 PIEIAQRITFEERVSVHNRGYLQKLVDD-----------------KLCLSYTQGSTTYSL

NRPA1 468 PPCFALKLTYPERVTPWNVEKLREAIINGPDIHPGATHYSDKSSTMKLPSTEKARRAIAR

C

NRPB1 379 PWSIALNLTYPETVTPYNIERLKELVDYGPHPPPGK-------TGAKYIIRDDGQRLDLR

NRPC1 384 PILMAQILTFPECVSRHNIEKLRQCVRNGPNKYPG---------ARNVRYPDGSSRTLVG

ScRpb1 367 PKSIAKTLTYPEVVTPYNIDRLTQLVRNGPNEHPG----------AKYVIRDSGDRIDLR

Active site

Dock

NRPD1 382 RLVAIQVNDLQTG---------DKIFRSLMDGDTVLMNRPPSIHQHSLIAMTVRILPTTS

NRPE1 385 RDGSKGHTELKPG---------QVVHRRVMDGDVVFINRPPTTHKHSLQALRVYVHE-DN

NRPA1 528 KLLSSRGATTELGKTCDINFEGKTVHRHMRDGDIVLVNRQPTLHKPSLMAHKVRVLKGEK

NRPB1 432 YLKKSSDQHLELG---------YKVERHLQDGDFVLFNRQPSLHKMSIMGHRIRIMP-YS

NRPC1 435 DYRKRIADELAIG---------CIVDRHLQEGDVVLFNRQPSLHRMSIMCHRARIMP-WR

D

ScRpb1 417 YSKRAGDIQLQYG---------WKVERHIMDNDPVLFNRQPSLHKMSMMAHRVKVIP-YS

Dock

Active site

*Metal A*

* * *

NRPD1 433 VVSLNPICCLPFRGDFDGDCLHGYVPQSIQAKVELDELVALDKQLINRQNGRNLLSLGQD

NRPE1 435 TVKINPLMCSPLSADFDGDCVHLFYPQSLSAKAEVMELFSVEKQLLSSHTGQLILQMGSD

NRPA1 588 TLRLHYANCSTYNADFDGDEMNVHFPQDEISRAEAYNIVNANNQYARPSNGEPLRALIQD

NRPB1 482 TFRLNLSVTSPYNADFDGDEMNMHVPQSFETRAEVLELMMVPKCIVSPQANRPVMGIVQD

NRPC1 485 TLRFNESVCNPYNADFDGDEMNMHVPQTEEARTEAITLMG--------------------

D

ScRpb1 467 TFRLNLSVTSPYNADFDGDEMNLHVPQSEETRAELSQLCAVPLQIVSPQSNKPCMGIVQD

Active site

Pore

NRPD1 493 SLTAAYLVNVEKNCYLNRAQMQQLQM-------------------------YCPFQLPPP

NRPE1 495 SLLSLRVM--LERVFLDKATAQQLAM-------------------------YGSLSLPPP

NRPA1 648 HIVSSVLL-TKRDTFLDKDHFNQLLFSSGVTDMVLSTFSGRSGKKVMVSASDAELLTVTP

NRPB1 542 TLLGCRKI-TKRDTFIEKDVFMNTLM----------------------WWEDFDGKVPAP

NRPC1 525 ------------DTFYDRAAFSLICS--------------------YMGDGMDSIDLPTP

E

ScRpb1 527 TLCGIRKL-TLRDTFIELDQVLNMLY----------------------WVPDWDGVIPTP

Pore

*Rpb8 Interaction*

NRPD1 528 AIIKASPSSTEPQWTGMQLFGMLFPPGFD-YTYPLNNVVV--------------------

NRPE1 528 ALRKSSKS--GPAWTVFQILQLAFPERLS-CKGDRFLVDG--------------------

NRPA1 707 AILKP-----VPLWTGKQVITAVLNQITKGHPPFTVEKATKLPVDFFKCRSREVKPNSGD

NRPB1 579 AILKP-----RPLWTGKQVFNLIIPKQINLLRYSAWHADTETG-----------------

NRPC1 553 TILKP-----IELWTGKQIFSVLLRPNASIRVYVTLNVKEKNFKKG--------------

E

ScRpb1 564 AIIKP-----KPLWSGKQILSVAIPNGIHLQRF-----DEGTT-----------------

Pore

NRPD1 567 ----------------------SNGELLSFSEGSAWLRD---------GEGNFIERLLKH

NRPE1 565 ------------------------SDLLKFDFGVDAMGS--------IINEIVTSIFLEK

NRPA1 762 LTKKKEIDESWKQNLNEDKLHIRKNEFVCGVIDKAQFAD----------YGLVHTVHELY

NRPB1 617 -----------FITPGDTQVRIERGELLAGTLCKKTLGT--------SNGSLVHVIWEEV

NRPC1 594 -----EHGFDETMCINDGWVYFRNSELISGQLGKATLALDIFPLGNGNKDGLYSILLRDY

ScRpb1 597 -----------LLSPKDNGMLIIDGQIIFGVVEKKTVGS--------SNGGLIHVVTREK

Pore

*Rpb2 Interaction*

NRPD1 596 DKGKVLDIIYSAQEMLSQWLLMRGLSVSLADLYLSSDLQSRKNLTEEISYGLREAEQVCN

NRPE1 593 GPKETLGFFDSLQPLLMESLFAEGFSLSLEDLSMSRADM--DVIHNLIIREISPMVSRLR

NRPA1 812 GSNAAGNLLSVFSRLFTVFLQTHGFTCGVDDLIILKDMD--EERTKQLQECENVGERVLR

NRPB1 658 GPDAARKFLGHTQWLVNYWLLQNGFTIGIGDTIADSSTM--EKINETISNAKTAVKDLIR

NRPC1 649 NSHAAAVCMNRLAKLSARWIGIHGFSIGIDDVQPGEELS--KERKDSIQFGYDQCHRKIE

ScRpb1 638 GPQVCAKLFGNIQKVVNFWLLHNGFSTGIGDTIADGPTM--REITETIAEAKKKVLDVTK

Pore

Funnel

*Rpb9 Interaction*

NRPD1 656 KQQLMVESWRDFLAVNGEDKEEDSVSDLARFCYERQKSATLSELAVSAFKDAYRDVQALA

NRPE1 651 LSYRDELQLEN----------------------------------------SIHKVKEVA

NRPA1 870 KTFGIDVDVQIDPQDM-RSRIERILYEDGESALASLDRSIVNYLNQCSSKGVMNDLLSDG

NRPB1 716 QFQGKELDPEP-----------------GRTMRDTFENRVNQVLNKAR-----DDAGSSA

NRPC1 707 EFNRGNLQLKA-----------------GLDGAKSLEAEITGILNTIR-----EATGKAC

ScRpb1 696 EAQANLLTAKH-----------------GMTLRESFEDNVVRFLNEAR-----DKAGRLA

Funnel

*Rpb9 Interaction*

NRPD1 716 YRYGDQSNSFLIMSKAGSKGNIGKLVQHSMCIGLQNSAVSLSFGFPRELTCAAWNDPNSP

NRPE1 671 ANFMLKSYSIRNLIDIKSNSAITKLVQQTGFLGLQLSDKKKFYTKTLVEDMAIFCKRKYG

NRPA1 929 LLKTPGRNCISLMTISGAKGSKVNFQQISSHLGQQDLEGKRVPRMVSGKTLPCFHPWDWS

F

NRPB1 764 QKSLAETNNLKAMVTAGSKGSFINISQMTACVGQQNVEGKRIPFGFDGRTLPHFTKDDYG

NRPC1 745 MSGLHWRNSPLIMSQCGSKGSPINISQMVACVGQQTVNGHRAPDGFIDRSLPHFPRMSKS

ScRpb1 734 EVNLKDLNNVKQMVMAGSKGSFINIAQMSACVGQQSVEGKRIAFGFVDRTLPHFSKDDYS

Funnel

*Rpb2 Interaction Bridge helix*

NRPD1 776 LRGAKGKDSTTTESYVPYGVIENSFLTGLNPLESFVHSVTSRDSSFSGNADLP--GTLSR

NRPE1 731 RISSSGDF----------GIVKGCFFHGLDPYEEMAHSIAAREVIVRSSRGLAEPGTLFK

NRPA1 989 PRAG--------------GFISDRFLSGLRPQEYYFHCMAGREGLVDTAVKTSRSGYLQR

NRPB1 814 PESR--------------GFVENSYLRGLTPQEFFFHAMGGREGLIDTAVKTSETGYIQR

F

NRPC1 805 PAAK--------------GFVANSFYSGLTATEFFFHTMGGREGLVDTAVKTASTGYMSR

ScRpb1 794 PESK--------------GFVENSYLRGLTPQEFFFHAMGGREGLIDTAVKTAETGYIQR

Funnel

Cleft

*Rpb5 Interaction*

NRPD1 834 RLMFFMRDIYAAYDGTVRNSFGNQLVQFTYETDGPVEDIT--------------------

NRPE1 781 NLMAVLRDIVITNDGTVRNTCSNSVIQFKYGVDSERGHQG--------------------

NRPA1 1035 CLMKNLESLKVNYDCTVRDADG-SIIQFQYGEDGVDVHRSS-------------------

NRPB1 860 RLVKAMEDIMVKYDGTVRNSLG-DVIQFLYGEDGMDAVWIESQKLDSLKMKKSEFDRTFK

NRPC1 851 RLMKALEDLLVHYDNTVRNASG-CILQFTYGDDGMDPALME-------------------

F

ScRpb1 840 RLVKALEDIMVHYDNTTRNSLG-NVIQFIYGEDGMDAAHIEKQSLDTIGGSDAAFEKRYR

Cleft

Foot

NRPD1 874 ------------------------------------------------------------

NRPE1 821 ------------------------------------------------------------

NRPA1 1075 ---------------------------------------------------FIEKFKELT

NRPB1 919 YEIDDENWNPTYLSDEHLEDLKGIRELRDVFDAEYSKLETDRFQLGTEIATNGDSTWPLP

NRPC1 891 ----------------------------------------------------GKDGAPLN

ScRpb1 899 VDLLNTDHTLDPSLLESGSEILGDLKLQVLLDEEYKQLVKDRKFLR-EVFVDGEANWPLP

Foot

Foot

NRPD1 874 ------------------------------------------------------------

NRPE1 821 ------------------------------------------------------------

NRPA1 1084 INQDMVLQKCSEDMLSG--------------ASSYIS------------------DLPIS

NRPB1 979 VNIKRHIWNAQKTFKIDLRKISDMHPVEIVDAVDKLQERLLVVPGDDALSVEAQKNATLF

NRPC1 899 FNRLFLKVQATCPPRSHHTYLS-------SEELSQKFEEELVRHDKSRVCTDAFVKSLRE

ScRpb1 958 VNIRRIIQNAQQTFHIDHTKPSDLTIKDIVLGVKDLQENLLVLRGKNEIIQNAQRDAVTL

*Rpb6 Interaction*

NRPD1 874 -------------------------------------------GEALGSLSACALSEAAY

NRPE1 821 ---------------------------------------LFEAGEPVGVLAATAMSNPAY

NRPA1 1112 LKKGAEKFVEAMPMNERIASKFVRQEELLKLVKSKFFASLAQPGEPVGVLAAQSVGEPST

NRPB1 1039 FNILLRSTLASKRVLEEYKLSREAFEWVIGEIESRFLQSLVAPGEMIGCVAAQSIGEPAT

G

NRPC1 952 FVSLLG--------------VKSASPPQVLYKASGVTDKQLEAGTAIGTIGAQSIGEPGT

ScRpb1 1018 FCCLLRSRLATRRVLQEYRLTKQAFDWVLSNIEAQFLRSVVHPGEMVGVLAAQSIGEPAT

G

Foot

Cleft

NRPD1 891 SALDQPISLLETSPLLNLKNVLECGSKKG-QREQTMSLYLSEYLSKKKHGFEYGSLEIKN

NRPE1 842 KAVLDSSPNSNSSWELMKEVLLCKVNFQNTTNDRRVILYLNECHCGKRFCQENAACTVRN

NRPA1 1172 QMTLNTFHLAGRGEMNVTLGIPRLQEILMTAAANIKTPIMTCPLLKG--KTKEDANDITD

NRPB1 1099 QMTLNTFHYAGVSAKNVTLGVPRLREIIN-VAKRIKTPSLSVYLTPEASKSKEGAKTVQC

NRPC1 998 QMTLKTFHFAGVASMNITQGVPRINEIIN-ASKNISTPVISAELEN--PLELTSARWVKG

ScRpb1 1078 QMTLNTFHFAGVASKKVTSGVPRLKEILN-VAKNMKTPSLTVYLEPGHAADQEQAKLIRS

Cleft

*Rpb9 Interaction*

NRPD1 950 HLEKLSFSEIVSTSMIIFSPSS-NTKVPLSPWVCHFHISEKVLKRKQLSAESVVSSLN--

NRPE1 902 KLNKVSLKDTAVEFLVEYRKQPTISEIFGIDSCLHGHIHLNKTLLQDWNISMQDIHQKCE

NRPA1 1230 RLRKITVADIIKSMELSVVPYTVYENEVCSIHKLKINLYKPEHYPKHTDITEEDWEETMR

NRPB1 1158 ALEYTTLRSVTQATEVWYDPDPMSTIIEEDFEFVRSYYEMPDEDVSP--DKISPWLLR--

NRPC1 1055 RIEKTTLGQVAESIEVLMTSTSASVRIILDNKIIEEACLS-----------ITPWSVKN-

ScRpb1 1137 AIEHTTLKSVTIASEIYYDPDPRSTVIPEDEEIIQLHFSLLDEEAEQSFDQQSPWLLR--

Cleft

Jaw

NRPD1 1007 ------------------EQYKSRNRELK-------------------------------

NRPE1 962 DVIN----------SLGQKKKKKATDDFK-------------------------------

NRPA1 1290 AVFLRKLEDAIETHMKMLHRIRGIHNDVTGPIAGNETDNDDSVSGKQNEDDGDDDGEGTE

NRPB1 1214 ---------------IELNREMMVDKKLS-------------------------------

NRPC1 1103 -------------SILKTPRIKLNDNDIR-------------------------------

ScRpb1 1195 ---------------LELDRAAMNDKDLT-------------------------------

Jaw

NRPD1 1018 ---LDIVDLDIQNTNHCSSDDQAMKDDNVCITVTVVEAS---------KHSVLELDAIRL

NRPE1 981 -----RTSLSVSECCSFRDPCGSKGSDMPCLTFSYNATDP---------DLERTLDVLCN

NRPA1 1350 VDDLGSDAQKQKKQETDEMDYEENSEDETNEPSSISGVEDPEMDSENEDTEVSKEDTPEP

NRPB1 1228 ---MADIAEKINLEFDDDLTCIFNDDNAQKLILRIRIMNDEGPKGELQDESAEDDVFLKK

NRPC1 1119 ---VLDTGLDITPVVD----------------------------------KSRAHFNLHN

ScRpb1 1209 ---MGQVGERIKQTFKNDLFVIWSEDNDEKLIIRCRVVR---PKSLDAETEAEEDHMLKK

Jaw

*Rpb9/Rpb2 Interaction*

NRPD1 1066 VLIPFLLDSPVKGDQGIKKVN---------------------------------------

NRPE1 1027 TVYPVLLEIVIKGDSRICSAN---------------------------------------

NRPA1 1410 QEESMEPQKEVKGVKNVKEQSKKKRRKFVRAKSDRHIFVKGEGEKFEVHFKFATDDPHIL

NRPB1 1285 IESNMLTEMALRGIPDINK-----------------------------------------

NRPC1 1142 LKN---------GIKTVER-----------------------------------------

ScRpb1 1263 IENTMLENITLRGVENIER-----------------------------------------

Jaw

Cleft

NRPD1 1087 --------------------------------ILWTDRPKAPKRNGNHLAGELYLKVTMY

NRPE1 1048 --------------------------------IIWNSSDMTTWIRNRHASRRGEWVLDVT

NRPA1 1470 LAQIAQQTAQKVYIQNSGKIERCTVANCGDPQVIYHGDNPKERREISNDEKKASPALHAS

NRPB1 1304 --------------------------------VFIKQVRKSRFDEEGGFKTSEEWMLDTE

NRPC1 1152 --------------------------------VVVAEDMDKSKQIDG----KTKWKLFVE

ScRpb1 1282 --------------------------------VVMMKYDRKVPSPTGEYVKEPEWVLETD

Cleft

*Rpb5 Interaction*

NRPD1 1115 GDRGKR----NCWTALLETCLPIMDMIDWGRSHPDNIRQCCSVYGIDAGRSIFVANLESA

NRPE1 1076 VEKSAVKQSGDAWRVVIDSCLSVLHLIDTKRSIPYSVKQVQELLGLSCAFEQAVQRLSAS

NRPA1 1530 G-------------VDFPALWEFQDKLDVRYLYSNSIHDMLNIFGVEAARETIIREINHV

NRPB1 1332 G-------------VNLLAVMCHED-VDPKRTTSNHLIEIIEVLGIEAVRRALLDELRVV

NRPC1 1176 G-------------TNLLAVMGTPG-INGRTTTSNNVVEVSKTLGIEAARTTIIDEIGTV

ScRpb1 1310 G-------------VNLSEVMTVPG-IDPTRIYTNSFIDIMEVLGIEAGRAALYKEVYNV

Cleft

NRPD1 1171 VSDTGKEILREHLLLVADSLSVTGEFVALNAKGWSKQRQVESTPAPFTQACFSSPSQCFL

NRPE1 1136 VRMVSKGVLKEHIILLANNMTCSGTMLGFNSGGYKALTRSLNIKAPFTEATLIAPRKCFE

NRPA1 1577 FKSYGISVSIRHLNLIADYMTFSGGYRPMSRMGGIA-----ESTSPFCRMTFETATKFIV

NRPB1 1378 ISFDGSYVNYRHLAILCDTMTYRGHLMAITRHGINR-----NDTGPLMRCSFEETVDILL

H

NRPC1 1222 MGNHGMSIDIRHMMLLADVMTYRGEVLGIQRTGIQK-----MDKSVLMQASFERTGDHLF

ScRpb1 1356 IASDGSYVNYRHMALLVDVMTTQGGLTSVTRHGFNR-----SNTGALMRCSFEETVEILF

Cleft

Clamp core

*Rpb2 Interaction Rpb6 Interaction*

NRPD1 1231 KAAKEGVRDDLQGSIDALAWGKVPGFGTGDQFEIIISPKVHGF-----------------

NRPE1 1196 KAAEKCHTDSLSTVVGSCSWGKRVDVGTGSQFELLWNQKETGL-----------------

NRPA1 1632 QAATYGEKDTLETPSARICLGLPALSGTGCFDLMQRVEL---------------------

NRPB1 1443 DAAAYAETDCLRGVTENIMLGQLAPIGTGDCELYLN-DEMLKNAIELQLPSYMDGLEFGM

NRPC1 1277 SAAASGKVDNIEGVTECVIMGIPMKLGTGILKVLQRTDDLPK-------------LKYGP

ScRpb1 1411 EAGASAELDDCRGVSENVILGQMAPIGTGAFDVMIDEESLVKYMPEQKITEIEDGQDGGV

H

Clamp core

NRPD1 1274 ------------------------------------------------------------

NRPE1 1239 ------------------------------------------------------------

NRPA1 1671 ------------------------------------------------------------

NRPB1 1492 TPARSPVSGTPYHEGMMSPNYLLSPNMRLSPMSDAQFSPYVGGMAFSPSSSPGYSPSSPG

NRPC1 1324 DPIIS-------------------------------------------------------

ScRpb1 1471 TPYSN-------ESGLVNADLDVKDELMFSPLVDSGSNDAMAG-GFTAYGGVDYG-----

NRPD1 1274 ------------------------------------------------------------

NRPE1 1239 ------------------------------------------------------------

NRPA1 1671 ------------------------------------------------------------

NRPB1 1552 YSPTSPGYSPTSPGYSPTSPGYSPTSPTYSPSSPGYSPTSPAYSPTSPSYSPTSPSYSPT

NRPC1 1329 ------------------------------------------------------------

Pol II heptad repeats

Pol II heptad repeats

Pol II heptad repeats

ScRpb1 1518 -EATSP----------FAAYGEAPTSPGFGVSSPGFSPTSPTYSPTSPAYSPTSPSYSPT

NRPD1 1274 ------------------------------------------------------------

NRPE1 1239 ------------------------------------------------------------

NRPA1 1671 ------------------------------------------------------------

NRPB1 1612 SPSYSPTSPSYSPTSPSYSPTSPSYSPTSPAYSPTSPAYSPTSPAYSPTSPSYSPTSPSY

NRPC1 1329 ------------------------------------------------------------

ScRpb1 1567 SPSYSPTSPSYSPTSPSYSPTSPSYSPTSPSYSPMSPSYSPTSPSYSPTSPSYSPTSPSY

NRPD1 1274 ------------------------------------------------------------

NRPE1 1239 ------------------------------------------------------------

NRPA1 1671 ------------------------------------------------------------

NRPB1 1672 SPTSPSYSPTSPSYSPTSPSYSPTSPAYSPTSPGYSPTSPSYSPTSPSYGPTSPSYNPQS

NRPC1 1329 ------------------------------------------------------------

ScRpb1 1627 SPTSPSYSPTSPSYSPTSPSYSPTSPAYSPTSPSYSPTSPSYSPTSPSYSPTSPSY----

NRPD1 1274 ------------------------------------------------------------

NRPE1 1239 ------------------------------------------------------------

NRPA1 1671 ------------------------------------------------------------

Pol II heptad repeats

NRPB1 1732 AKYSPSIAYSPSNARLSPASPYSPTSPNYSPTSPSYSPTSPSYSPSSPTYSPSSPYSSGA

NRPC1 1329 ------------------------------------------------------------

ScRpb1 1683 ----------------------SPTSPNYSPTSPSYSPTSPGYSPGSPAYSP--------

NRPD1 1274 ------------------------------------------------------------

NRPE1 1239 -------------------------------------------DDKEETDVYSFLQMVIS

NRPA1 1671 ------------------------------------------------------------

NRPB1 1792 SPDYSPSAGYSPTLPGYSPSSTGQYTPHEGDKKDKTGKKDASKDDKGNP-----------

NRPC1 1329 ------------------------------------------------------------

ScRpb1 1713 -------------------------------KQDEQ-KHNENENSR--------------

NRPD1 1274 ------------------------------------------------------------

NRPE1 1256 TTNADAFVSSPGFDVTEEEMAEWAESPERDSALGEPKFEDSADFQNLHDEGKPSGANWEK

NRPA1 1671 ------------------------------------------------------------

NRPB1 1841 ------------------------------------------------------------

NRPC1 1329 ------------------------------------------------------------

ScRpb1 1727 ------------------------------------------------------------

NRPD1 1274 --------------------------------TTPVDVYDLLSSTKTMRRTNSAPKSDK-

NRPE1 1316 SSSWDNGCSGGSEWGVSKSTGGEANPESNWEKTTNVEKEDAWSSWNTRKDAQESSKSDSG

NRPA1 1671 ------------------------------------------------------------

NRPB1 1841 ------------------------------------------------------------

NRPC1 1329 ------------------------------------------------------------

ScRpb1 1727 ------------------------------------------------------------

NRPD1 1301 ------------------------------------------------------------

Pol V repeats

NRPE1 1376 GAWGIKTKDADADTTPNWETSPAPKDSIVPENNEPTSDVWGHKSVSDKSWDKKNWGTESA

NRPA1 1671 ------------------------------------------------------------

NRPB1 1841 ------------------------------------------------------------

NRPC1 1329 ------------------------------------------------------------

ScRpb1 1727 ------------------------------------------------------------

NRPD1 1301 -------------------------------------------ATVQPFGLLHS------

Pol V repeats

NRPE1 1436 PAAWGSTDAAVWGSSDKKNSETESDAAAWGSRDKNNSDVGSGAGVLGPWNKKSSETESNG

NRPA1 1671 ------------------------------------------------------------

NRPB1 1841 ------------------------------------------------------------

Pol V repeats

NRPC1 1329 ------------------------------------------------------------

ScRpb1 1727 ------------------------------------------------------------

NRPD1 1312 ------------------------------------------------------------

NRPE1 1496 ATWGSSDKTKSGAAAWNSWDKKNIETDSEPAAWGSQGKKNSETESGPAAWGAWDKKKSET

NRPA1 1671 ------------------------------------------------------------

NRPB1 1841 ------------------------------------------------------------

NRPC1 1329 ------------------------------------------------------------

ScRpb1 1727 ------------------------------------------------------------

NRPD1 1312 ------------------------------------------------------------

Pol V repeats

NRPE1 1556 EPGPAGWGMGDKKNSETELGPAAMGNWDKKKSDTKSGPAAWGSTDAAAWGSSDKNNSETE

NRPA1 1671 ------------------------------------------------------------

NRPB1 1841 ------------------------------------------------------------

NRPC1 1329 ------------------------------------------------------------

ScRpb1 1727 ------------------------------------------------------------

NRPD1 1312 ----------------------------------------------AFLKDIKVLDGK--

Pol V repeats

NRPE1 1616 SDAAAWGSRNKKTSEIESGAGAWGSWGQPSPTAEDKDTNEDDRNPWVSLKETKSREKDDK

NRPA1 1671 ------------------------------------------------------------

NRPB1 1841 ------------------------------------------------------------

NRPC1 1329 ------------------------------------------------------------

ScRpb1 1727 ------------------------------------------------------------

NRPD1 1324 -----GIPMSLLRTIFTWKN----------------------------------------

NRPE1 1676 ERSQWGNPAKKFPSSGGWSNGGGADWKGNRNHTPRPPRSEDNLAPMFTATRQRLDSFTSE

NRPA1 1671 ------------------------------------------------------------

NRPB1 1841 ------------------------------------------------------------

NRPC1 1329 ------------------------------------------------------------

ScRpb1 1727 ------------------------------------------------------------

NRPD1 1339 -------IELLSQSLKRILHSYEIN---ELLNERDEGLVKMVLQLHPNSVEKIGPGVKGI

DeCL-like domain

NRPE1 1736 EQELLSDVEPVMRTLRKIMHPSAYPDGDPISDDDKTFVLEKILNFHPQKETKLGSGVDFI

NRPA1 1671 ------------------------------------------------------------

NRPB1 1841 ------------------------------------------------------------

NRPC1 1329 ------------------------------------------------------------

ScRpb1 1727 ------------------------------------------------------------

DeCL-like domain

NRPD1 1389 RVAKS-KHGDSCCFEVVRIDGTFEDFSYHKCVLGATKIIAPKKMNFYKSKYLKN----GT

NRPE1 1796 TVDKHTIFSDSRCFFVVSTDGAKQDFSYRKSLNNYLMKKYPDRAEEFIDKYFTKPRPSGN

NRPA1 1671 ------------------------------------------------------------

NRPB1 1841 ------------------------------------------------------------

NRPC1 1329 ------------------------------------------------------------

ScRpb1 1727 ------------------------------------------------------------

QS-rich domain

NRPD1 1444 LESGGFSENP--------------------------------------------------

NRPE1 1856 RDRNNQDATPPGEEQSQPPNQSIGNGGDDFQTQTQSQSPSQTRAQSPSQAQAQSPSQTQS

NRPA1 1671 ------------------------------------------------------------

NRPB1 1841 ------------------------------------------------------------

NRPC1 1329 ------------------------------------------------------------

ScRpb1 1727 ------------------------------------------------------------

NRPD1 1454 ------------------------------------------------------------

QS-rich domain

NRPE1 1916 QSQSQSQSQSQSQSQSQSQSQSQSQSQSQSQSPSQTQTQSPSQTQAQAQSPSSQSPSQTQ

NRPA1 1671 ------------------------------------------------------------

NRPB1 1841 ------------------------------------------------------------

NRPC1 1329 ------------------------------------------------------------

ScRpb1 1727 ------------------------------------------------------------

NRPD1 1454 -

NRPE1 1976 T

NRPA1 1671 -

NRPB1 1841 -

NRPC1 1329 -

ScRpb1 1727 -
