# Supplementary figures and images for: Metal A and Metal B Sites of Nuclear RNA Polymerases Pol IV and Pol V Are Required for siRNA-Dependent DNA Methylation and Gene Silencing
Source: PLoS One. 2009 Jan 1;4(1):e4110. doi: 10.1371/journal.pone.0004110 (PMC2605557; doi:10.1371/journal.pone.0004110)

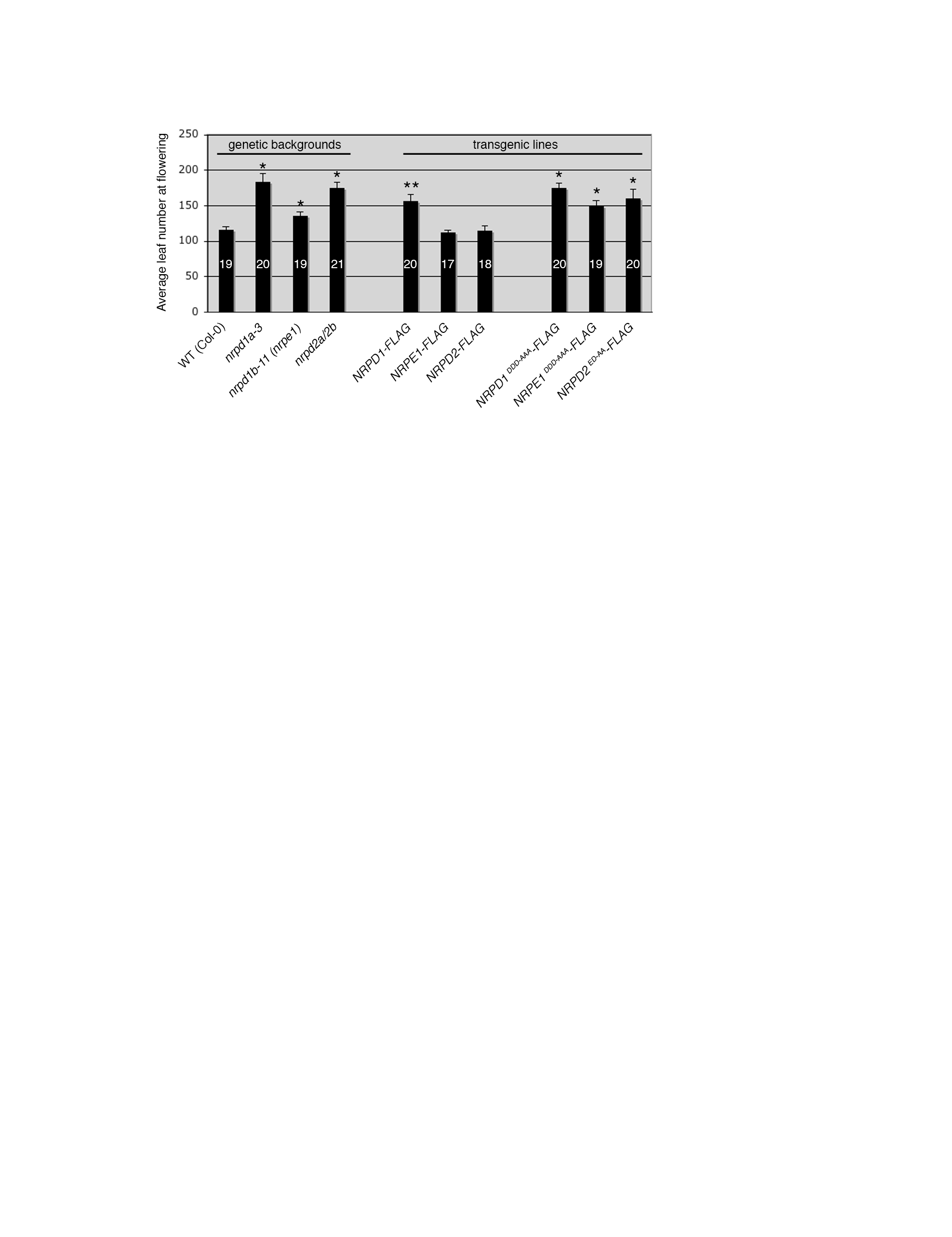

Supplement: Figure S2 — Flowering time control is dependent upon the Pol IV and Pol V active sites. nrpd1a, nrpe1/nrpd1b and nrpd2 mutants, or transgenic lines generated by transforming these mutants with wild-type or active site mutant versions of NRPD1, NRPE1/NRPD1b or NRPD2a full-length transgenes, were grown side-by-side under short day conditions (8 hours light/16 hours dark). The positions of pots were changed every 4–6 days according to a randomized plot design. The total number of rosette leaves for each plant was counted when the bolt (flower stalk) achieved a height of 5 cm. The histograms show the average number of leaves at flowering+/−the standard error of the mean. Asterisks denote mean values that are significantly different (p<0.05) from the wild-type (WT; ecotype Col-0) control population as determined by using the Student t-Test; a double asterisk denotes a value that is significantly different from both the WT and nrpd1a-3 controls. The number of individual plants analyzed for each genotype is denoted by the numeric value inside each vertical bar. As expected, based on prior studies [1], [2], nrpd1a-3, nrpd1b-11 (nrpe1) and nrpd2 mutant plants were significantly delayed in flowering relative to wild-type plants. Flowering time of the mutants was unaffected by transforming them with the NRPD1, NRPE1 or NRPD2 active site mutant transgenes. However, wild-type flowering time was restored by the non-mutant NRPE1-FLAG or NRPD2-FLAG transgenes. It is noteworthy that the non-mutant NRPD1-FLAG transgene did not fully restore flowering time in the nrpd1a-3 mutant background to that of wild-type plants, perhaps reflecting the incomplete rescue of siRNA levels shown in Figure 2B. (0.45 MB TIF) [file pone.0004110.s004.tif]
